# Examination and characterisation of the effect of amitriptyline therapy for chronic neuropathic pain on neuropeptide and proteomic constituents of human cerebrospinal fluid

**DOI:** 10.1016/j.bbih.2020.100184

**Published:** 2020-12-07

**Authors:** Jonathan Royds, Hilary Cassidy, Melissa J. Conroy, Margaret R. Dunne, Joanne Lysaght, Connail McCrory

**Affiliations:** a Department of Pain Medicine, St. James Hospital, Dublin and School of Medicine, Trinity College Dublin, Ireland; b Systems Biology Ireland, School of Medicine, University College Dublin, Dublin 4, Ireland; c Department of Surgery, Trinity Translational Medicine Institute, St. James’s Hospital and Trinity College Dublin, Dublin 8, Ireland; d Trinity St James’s Cancer Institute, St James’s Hospital Dublin, Dublin 8, Ireland

**Keywords:** Amitriptyline, Mechanism of action, Cerebrospinal fluid, Neuropathic pain, Neuroimmune, Neuropharmacology

## Abstract

**Introduction:**

Amitriptyline is prescribed to reduce the intensity of chronic neuropathic pain. There is a paucity of validated *in vivo* evidence in humans regarding amitriptyline’s mechanism of action. We examined the effect of amitriptyline therapy on cerebrospinal fluid (CSF) neuropeptides and proteome in patients with chronic neuropathic pain to identify potential mechanisms of action of amitriptyline.

**Methods:**

Patients with lumbar radicular neuropathic pain were selected for inclusion with clinical and radiological signs and a >50% reduction in pain in response to a selective nerve root block. Baseline (pre-treatment) and 8-week (post-treatment) pain scores with demographics were recorded. CSF samples were taken at baseline (pre-treatment) and 8 weeks after amitriptyline treatment (post-treatment). Proteome analysis was performed using mass spectrometry and secreted cytokines, chemokines and neurotrophins were measured by enzyme-linked immunosorbent assay (ELISA).

**Results:**

A total of 9/16 patients experienced a >30% reduction in pain after treatment with amitriptyline and GO analysis demonstrated that the greatest modulatory effect was on immune system processes. KEGG analysis also identified a reduction in PI3K-Akt and MAPK signalling pathways in responders but not in non-responders. There was also a significant decrease in the chemokine eotaxin-1 (p ​= ​0.02) and a significant increase in the neurotrophin VEGF-A (p ​= ​0.04) in responders.

**Conclusion:**

The CSF secretome and proteome was modulated in responders to amitriptyline verifying many pre-clinical and *in vitro* models. The predominant features were immunomodulation with a reduction in pro-inflammatory pathways of neuronal-glia communications and evidence of a neurotrophic effect.

## Highlights

• The proteome and secretome of cerebrospinal fluid is modulated with amitriptyline.
• Immune system process is a key mechanism for amitriptyline in neuropathic pain.
• Responders to amitriptyline show a reduction in MAPK and PI3K-Akt pathways.
• A pain reduction is associated with a significant reduction of eotaxin in CSF.
• Amitriptyline likely has a neurotrophic effect in responders with neuropathic pain.

## 1 Introduction

Amitriptyline is a tertiary amine, tricyclic antidepressant first introduced in 1961 (Fangmann et al., 2008). Amitriptyline’s mechanism of action in the treatment of depression include re-uptake inhibition of serotonin and noradrenaline at the synaptic cleft (Hyttel et al., 1980). The pharmacodynamics of amitriptyline proposed from pre-clinical and *in vitro* studies are extensive but many have not been validated by *in vivo* evidence in humans (Couch and Amitriptyline Versus Placebo Study G, 2011; Freysoldt et al., 2009; Guan et al., 2019; Himmerich et al., 2010; Hisaoka et al., 2011; Hisaoka-Nakashima et al., 2016; Hutchinson et al., 2010; Jang et al., 2009; Jeanson et al., 2016; Kajitani et al., 2012; Kim et al., 2019; Kremer et al., 2016; Kremer et al., 2018; Lawson, 2017; Moore et al., 2015; Obuchowicz et al., 2006; Paumier et al., 2015; Rambe et al., 2015; Tai et al., 2006; Valera et al., 2014; van den Driest et al., 2017; Wang et al., 2004; Wolff et al., 2016). For this reason, amitriptyline’s mechanism of action in many off-label applications including neuropathic pain, fibromyalgia, migraine prophylaxis and complex regional pain syndrome (CRPS) have yet to be defined. Amitriptyline’s analgesic effect is achieved using lower doses than is required to treat depression and there are reports of a faster onset of action for alleviation of pain (Moore et al., 2015; Onghena and Van Houdenhove, 1992). This suggests that at different concentrations amitriptyline targets different pathways and may have a different mechanism of action for off-label applications including chronic neuropathic pain (CNP).

The pathophysiology of neuropathic pain and other neuroinflammatory conditions has been attributed at least in part to pathological changes in the neuroimmune interface (Albrecht et al., 2018; Duffy et al., 2018; Grace et al., 2014; Ji et al., 2018; Kothur et al., 2016; Loggia et al., 2015; Luchting et al., 2015; Royds and McCrory, 2018). This involves a multi-directional communication between neurons, immune cells and glia (Grace et al., 2014; Talbot et al., 2016). Many of the mechanisms proposed for the therapeutic action of amitriptyline in CNP relate to pathways within this interface (Himmerich et al., 2010; Hisaoka et al., 2011; Hisaoka-Nakashima et al., 2016; Hutchinson et al., 2010; Jang et al., 2009; Jeanson et al., 2016; Kajitani et al., 2012; Kim et al., 2019; Lawson, 2017; Obuchowicz et al., 2006; Paumier et al., 2015; Rambe et al., 2015; Tai et al., 2006; Valera et al., 2014). *In vitro* studies have demonstrated amitriptyline’s pharmacodynamic effect on glial cells, which are the predominant cells within the central nervous system with many anti-inflammatory mechanisms described (Hisaoka et al., 2011; Hutchinson et al., 2010; Jeanson et al., 2016; Kajitani et al., 2012; Obuchowicz et al., 2006; Valera et al., 2014). Specifically, amitriptyline has reduced pro-inflammatory cytokines and suppressed ERK 1/2 and MAPK signalling proteins associated with an increase in mechanical withdrawal threshold in mice (Kim et al., 2019). Amitriptyline has potential neurotrophic activity as well, inducing dynamic changes in brain derived neurotrophic factor (BDNF) (Hisaoka-Nakashima et al., 2016; Paumier et al., 2015), and glial cell derived neurotrophic factor (GDNF) *in vitro* (Baranov et al., 2014; Hisaoka et al., 2011; Hisaoka-Nakashima et al., 2015; Jang et al., 2009; Paumier et al., 2015). Tricyclic compounds have also demonstrated upregulation of vascular endothelial growth factor (VEGF) in the hippocampus of rodents (Greene et al., 2009).

*In vitro* studies of human T cells have demonstrated anti-inflammatory properties of amitriptyline by reducing the frequency of IFN-γ producing CD8^+^ cells and IL-17 producing CD8^+^ and CD4^+^ cells (Royds et al., 2020b). Tricyclic antidepressants have also demonstrated prevention of differentiation of monocytes into macrophages *in vitro* (Ying et al., 2002). CNP conditions including HIV neuropathy (Ho et al., 2013; Shi et al., 2012; Wang et al., 2014), diabetic neuropathy (Tang et al., 2013; Totsch and Sorge, 2017) and chronic radicular pain (Albrecht et al., 2018; Totsch and Sorge, 2017) have all implicated neuroimmune dysfunction in their pathophysiology. Although not effective in every case, amitriptyline remains a first line medication for patients with these conditions (Colloca et al., 2017; Finnerup et al., 2015).

Lumbar/sacral radicular pain is neuropathic pain radiating down one or more lumbar/sacral dermatomes. This pain is also described commonly as ‘sciatica’ or ‘nerve root pain’. The point prevalence is 4.6–13.4% and lifetime prevalence is 1.2%–43%, which means it is the most common form of neuropathic pain (Dworkin et al., 2007). Acute pain becomes chronic in approximately 30% of patients (Van Boxem et al., 2010). Diagnosis is made based on history, physical examination and radiological evaluation with confirmation provided by a diagnostic nerve root block (Van Boxem et al., 2010). Amitriptyline is frequently the first therapy employed to treat chronic radicular pain (Gelijkens et al., 2014; Vanelderen et al., 2015). A randomised controlled trial demonstrated amitriptyline was superior to placebo in patients with sub-acute lumbar radicular pain (Gelijkens et al., 2014). We hypothesised, assuming a central mechanism of action of amitriptyline, that it modulates the neuroimmune interface alleviating the symptoms of CNP. The examination of neuropeptide and proteomic constituents of cerebrospinal fluid (CSF) has previously been utilised to explore the mechanisms of action of therapies (Das et al., 2018; Lind et al., 2016; McCarthy et al., 2013; McCarthy and McCrory, 2014; Royds and McCrory, 2018). We examined and characterised the cytokine networks and proteomic constituents of CSF before and after amitriptyline treatment using lumbar radicular pain as a clinical model to identify the mechanistic actions of amitriptyline and provide information regarding the pathophysiology of CNP.

## 2 Methods

### 2.1 Location/ethics/registration

This was a prospective interventional observational study performed in St James’s Hospital, Dublin 8, Ireland; a tertiary referral centre for chronic pain. Ethical approval from the St James’s and AMNCH Research Ethics Committee, Dublin, Ireland was sought and obtained. The study was registered online at http://www.isrctn.com/ISRCTN70120536.

### 2.2 Participants

Patients attending the pain clinic at St. James Hospital, Dublin were offered inclusion if they met the following inclusion/exclusion criteria. The inclusion criteria were: patients aged 20–65 years with lumbar radicular pain for >6 months, clinical and radiological evidence of lumbar radicular pain, Douleur Neuropathique (DN4) score of >3 and a reduction in Numerical Pain score (NRS) of >50% after diagnostic nerve root block. Exclusion criteria were: patient refusal, central spinal stenosis, anticoagulant medication, infection, pregnancy, breastfeeding, corticosteroid therapy or NSAID’s, stroke, psychiatric history, history of ischaemic heart disease, arrhythmia or heart block, cerebral impairment, current anti-neuropathic medication (excluding opioids) or biologic medication. All patients were given an information leaflet about inclusion in the study. All patients signed a consent form approved by the hospital ethics committee for inclusion in the study. A consent form for the lumbar puncture (CSF sampling) and selective nerve root block was also signed.

### 2.3 CSF sampling

Under strict asepsis and AAGBI guidelines (Association of Anaesthetists of Great et al., 2014), CSF was obtained between the fourth and fifth lumbar vertebra under fluoroscopic guidance. This occurred at the same time period between 13:00–14:00 ​h with the patients fasting for 13–14 ​h prior to sample collection. Lidocaine 1%, 2–3 ​ml was infiltrated at the skin for analgesia. An introducer and 25 Gauge Whittacre needle (B braun®) was inserted until resistance entering the dura was felt. 1 ​ml of CSF (2 ​ml in total) was collected in two separate tubes: one for ELISA and one for mass spectrometry. The acquired CSF samples were visually inspected for blood contamination. The proteomics aliquots were centrifuged for 10 ​min at 2000 g and the supernatant was transferred to a new tube. The tubes were immediately frozen at −20 ​°C for ELISA and at −80 ​°C for proteomics. A second consented lumbar puncture (LP) sample was obtained in the same manner after 8 weeks. The time period of 8 weeks was selected to be consistent with the treatment course of anti-neuropathic medications recommended in order to gauge efficacy (Finnerup et al., 2015).

### 2.4 Pain measurement and diagnosis

Each patient completed an average 24-h (NRS) (Hawker et al., 2011) and a DN4 score (Bouhassira et al., 2005) by prior to obtaining the initial CSF sample. After CSF sampling the patients underwent a selective nerve root block. The patients were placed in prone position with pillows to diminish lumbar lordosis. Under strict asepsis and fluoroscopy the vertebra with the corresponding affected nerve was levelled off and rotated to the ipsilateral side so the spinous processes were in line with the contralateral facet column. A 22 Gauge needle was placed inferior to the pedicle and advanced under lateral fluoroscopic guidance to the posterior “safe line” of the epidural space. Iohexol 240 ​mg ​l/ml (Omnipaque TM, GE Healthcare, Cork, Ireland) 0.5 ​ml was injected to confirm spread along the distribution of the nerve and rule out intravascular placement of the needle. Once in the correct position, 1 ​ml of 1% lidocaine was injected. Patients were again asked to complete a NRS pain score 30 ​min after the diagnostic block. Successful block was determined if the decrease in NRS pain score was >50%.

### 2.5 Intervention

Patients treatment with amitriptyline 10 ​mg nocte was initiated (at night) following the first sample collection. The patients had the option of ceasing the medication and withdrawing from the study at any time. If this occurred, they would have their baseline (pre-treatment) sample included in the analysis, but a second CSF sample would not be taken. The patients were asked to remain on their other medications including opioids until after the second CSF sample. After one month if tolerated the dose was increased to 25 ​mg. After 8 weeks the patient returned for the second CSF sample with repeat NRS and DN4 scores recorded. Following completion of the study the patients were given the option of staying on the medication or not. Their answer and reason were also recorded. Successful treatment with amitriptyline was determined by having a >30% reduction in NRS at 8 weeks.

### 2.6 Quantification of soluble mediators in CSF

Glial Cell Derived Neurotrophic factor (GDNF) and Fractalkine singleplex ELISAs (Abcam, Cambridge, UK) were carried out according to the manufacturer’s guidelines. Mesoscale Discovery (MSD, Rockville, MD, USA) V-Plex Human Cytokine 30-Plex kit, R-Plex Human Brain Derived Neurotrophic factor (BDNF) antibody set with MSD Gold 96-plate pack and 96- well 4-spot prototype human Nerve Growth Factor (NGF) ELISAs were also carried out according to the manufacturer’s instructions. MSD plates were read using MesoScale Diagnostics Sector S600. The sensitivities to the kits are available at www.mesoscale.com, www.abcam.com and in our recent published work (Royds et al., 2020a).

### 2.7 Sample preparation and protein identification for mass spectrometry

All mass spectrometry (MS) and assistance with data analysis was performed by Dr Hilary Cassidy, Systems Biology Ireland, UCD (University College Dublin). Sample preparation and protein identification have previously been described (Royds et al., 2020a). SP3 preparation was performed according to the protocol of Hughes and colleagues (Hughes et al., 2014). The SP3 protocol utilizes commercially available beads which carry a carboxylate moiety. For this experiment both hydrophobic and hydrophilic Sera-Mag Speed bead Magnetic carboxylate modified particles were employed in a 1:1 mix (GE Healthcare). Prior to use, the beads were combined in a ratio of 1:1 (v/v), rinsed and reconstituted in MS grade water (Fisher Scientific) at a stock concentration of 10 ​μg/ml and stored at 4 ​°C until required.

SP3 preparation was performed according to the protocol of Hughes et al. (2014). Briefly, 200 ​μg CSF was resuspended in 100 ​μl lysis buffer [6 ​M urea, 2 ​M thiourea, 50 ​mM MOPS) and centrifuged for 15 ​min at 15,000 RCF at 4 ​°C to remove any cellular debris. The supernatant was transferred to a fresh Eppendorf tube. The CSF was reduced by adding 0.2 ​M 1,4-dithiothreitol (DTT; Sigma Aldrich) and incubated at 37 ​°C on a shaker at 700 ​rpm for 15 ​min. Samples were then alkylated by adding 0.4 ​M iodoacetamide (IAA; Sigma Aldrich). Next acetonitrile (ACN; Sigma Aldrich) was added to each sample to give a final concentration of 70% acetonitrile (v/v) and the prepared SP3 bead mixture was added to each sample and rotated for 18 ​min at room temperature. Subsequently the beads were immobilized by incubation for 2 ​min on the DynaMag-2™ stand (Thermo Fisher). The supernatant was discarded and the pellet was rinsed with 70% (v/v) ethanol in water and 100% ACN. Beads were resuspended in 50 ​mM ammonium bicarbonate (NH_4_HCO_3_; Sigma Aldrich). Lyophilised sequence grade trypsin (Promega) was resuspended in 50 ​mM ammonium bicarbonate to a final concentration of 0.5 ​μg/μl and the pH was adjusted to pH 7 before 4 ​μl of trypsin was added to each sample. After overnight digestion at 37 ​°C on a thermoshaker at 500 ​rpm, an additional 8 ​μl of prepared bead mixture was added to the samples and ACN was added to reach a final concentration of 95% (v/v). After mixing and incubation, the supernatant was removed and beads were rinsed with 100% ACN. The peptides bound to the beads were eluted using HPLC grade water with intermittent vortexing. The supernatant containing the purified peptides was transferred into a fresh tube containing 2 ​μl of 10% acetic acid. The samples were placed on the DynaMag-2™ for 5 ​min before the supernatant was transferred to MS vials for analysis.

### 2.8 LC-MS/MS analysis

Each sample was run in duplicate on a Thermo Scientific Q Exactive mass spectrometer connected to a Dionex Ultimate 3000 (RSLCnano) chromatography system. Each sample was loaded onto a fused silica emitter (75 ​μm ID), pulled using a laser puller (Sutter Instruments P2000, Novato, CA, USA), packed with ReprocilPur (Dr Maisch, Ammerbuch-Entringen, Germany) C18 (1.9 ​μm; 12 ​cm in length) reverse phase media and were separated by an increasing acetonitrile gradient over 60 ​min at a flow rate of 250 ​nL/min direct into a Q-Exactive MS. The MS was operated in positive ion mode with a capillary temperature of 320 ​°C, and with a potential of 2300 ​V applied to the frit. All data was acquired while operating in automatic data dependent switching mode. A high resolution (70,000) MS scan (300–1600 m/z) was performed using the Q Exactive to select the 12 most intense ions prior to MS/MS analysis using high-energy collision dissociation (HCD).

### 2.9 Data analysis and statistics

The ELISA statistical analysis was performed using Prism Graph Pad version 8.0. Fishers exact test was used to compare categorical data. Non-parametric paired and unpaired tests were used where appropriate, Wilcoxon Sign Rank and Mann Whitney respectively for continuous data. Data was expressed in means with standard error of means (SEM). Correlations between the percentage reduction in pain and the difference in the concentration of neuropeptides before and after amitriptyline were calculated using Spearman test with r, confidence intervals (CI) and p values. P values of <0.05 were considered to be significant.

For proteomics, proteins were identified and quantified by MaxLFQ (Cox et al., 2014) by searching with MaxQuant version 1.5 against the Homo Sapiens reference proteome database which was obtained from Uniprot. Normalisation is conducted through the MaxQuant LFQ algorithm for label-free quantification (Cox et al., 2014), which has successfully been benchmarked against other software solutions for label-free quantification, independently confirming its performance. MaxLFQ is a generic method for label-free quantification that can be combined with standard statistical tests of quantification accuracy for each of thousands of quantified proteins (Weisser et al., 2013). In brief, protein abundance profiles are assembled using the maximum possible information from MS signals, given that the presence of quantifiable peptides varies from sample to sample. This is based on the assumption that most proteins do not or only minimally change between conditions, to have a constant baseline [the algorithm still works with (quantitative) changes in about one third of all proteins] (Cox et al., 2014). Once the Maxquant analysis is complete, the individual LFQ intensities for all technical replicates were expressed as a Log_2_ value and an average Log_2_ value was determined for each of the treatment groups, i.e. responders and non-responders.

For the purposes of identifying proteins which were significantly altered following amitriptyline in the responders and non-responders, strict filtering settings were applied to the proteomics data in order identify proteins which were significantly increased [log fold change (LFC) ​> ​2, FDR< 0.05] and decreased (LFC ​< ​−2, FDR ​< ​0.05) using Log(p) ​> ​1.13 as a cut off following amitriptyline.

Proteins found to be differentially expressed between groups were subjected to pathway mapping analysis and were distributed into categories according to their cellular component, molecular function, and biological process using Ingenuity Pathway Analysis (IPA) [QIAGEN (Redwood City, CA)] or STRING Database (Version 10.5). STRING (www.string-db.org) was used to generate protein-protein interaction networks, which were then imported into Cytoscape for further editing (Version 3.4.0). The NeuroPep database (islab.info/NeuroPep/) and the neuropeptides database (www.neuropeptides.nl) were employed to identify neuropeptides from mass spectrometry. Kyoto Encyclopedia of Genes and Genomes (Kegg) pathway analysis was used to determine increased and decreased expression of proteins.

## 3 Results

### 3.1 Patient related outcomes

**Fig. 1:**
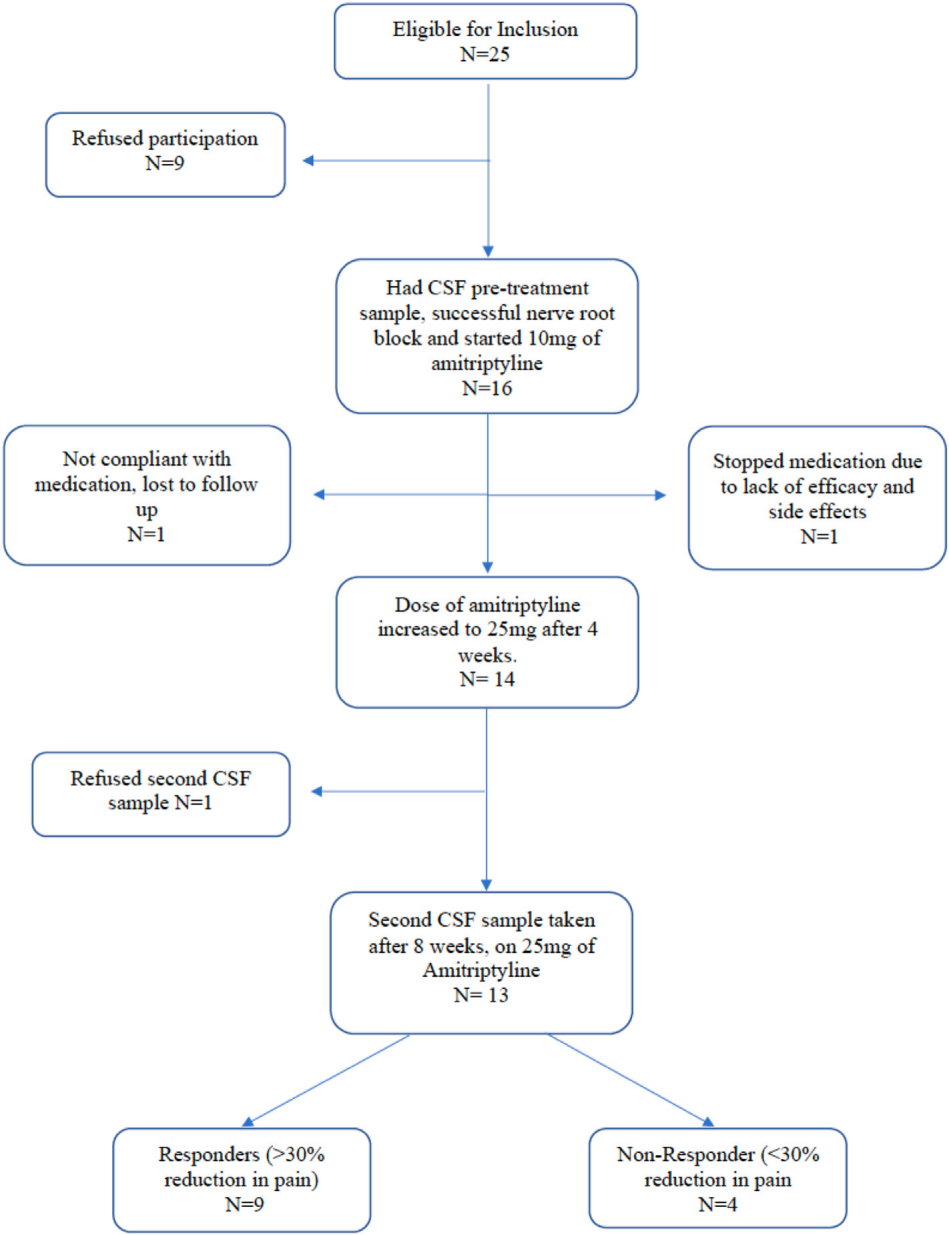
Patient flow and Consort diagram of patients eligible for inclusion for the study, intervention and Cerebrospinal fluid (CSF) sampling.

A total of 16 patients entered the study and had a CSF sample taken prior to commencing amitriptyline (pre-treatment sample) (Fig. 1). The demographics of the patients including their opioid medications are summarised in Table 1. All patients reported a successful diagnostic nerve root block with a >50% reduction in pain according to NRS and were started on 10 ​mg of amitriptyline (Fig. 1, Table 2). One patient reported a lack of efficacy with amitriptyline and problematic anticholinergic side effects at 3 weeks and ceased the medication (Study ID 103). Another patient reported a lack of efficacy with amitriptyline and poor therapeutic regime compliance and was subsequently lost to follow up (Study ID 104) (Fig. 1). Fourteen patients (14/16, 87.5%) achieved a dose escalation to 25 ​mg after 4 weeks of treatment (Fig. 1). Thirteen patients (13/16, 81%) had a second sample of CSF taken, one patient refused a second CSF sample and was a non-responder to amitriptyline (Study ID 106). In total, there were 16 CSF samples before amitriptyline (pre-treatment samples) and 13 samples after an 8-week course of amitriptyline (post-treatment samples). Nine patients reported a >30% reduction in pain according to NRS after 8 weeks of amitriptyline (9/16, 56%). We performed analysis on the patients who responded to amitriptyline by reporting a >30% reduction in NRS at 8 weeks (n ​= ​9), classified as ‘responders’. We also performed analysis on the ‘non-responders’ (<30% reduction in pain) as a comparative group (n ​= ​7), this included 7 “pre-treatment” and 4 “post-treatment” samples. The patients who did not have a second CSF sample taken had their pre-treatment sample included in the analysis on an intention to treat basis, all were non-responders. Patients 103 and 106 had pain scores taken at 8 weeks. No pain score was taken at 8 weeks in patient 104 but there was no reported benefit to the medication before being lost to follow up. There was no difference in demographics, opioid use and pre-treatment neuropeptide concentrations within the CSF of the responder group and the non-responder group (Table 3). Out of the patients that started the study, 10/16 (62.5%) remained on amitriptyline after 8 weeks. The most common reasons given for remaining on amitriptyline were due to pain reduction and improved sleep (Table 2). There was no dose escalation or de-escalation of opioids recorded in any of the patients.

**Table 1:** Summary of patient demographics including age, gender, nerve root anatomical location of radicular pain, opioids and morphine milligram equivalents (MME).

Study ID	Age	Sex	Affected Nerve Root	Opioid Medication	Type and Dose of Opioid	Morphine milligram equivalents (MME)
101^a^	63	Female	L5	No	–	–
102^a^	48	Male	L5	No	–	–
103	61	Female	L5	No	–	–
104	35	Male	S1	No	–	–
105^a^	56	Male	L5	Yes	Oxycodone 60 ​mg	120 ​mg
106	57	Male	L5/S1	No	–	–
107^a^	64	Male	L5	Yes	Fentanyl patch 75 mcg/hr	270 ​mg
108^a^	45	Female	L5	Yes	Oxycodone 20 ​mg	40 ​mg
109	50	Female	L5	Yes	Tramadol 100 ​mg	20 ​mg
110	30	Male	L5/S1	No	–	–
111^a^	53	Female	L4	Yes	Codeine 240 ​mg	24 ​mg
112	40	Female	L5	No	–	–
113^a^	64	Female	L5	No	–	–
114^a^	45	Female	L5	Yes	Tramadol 200 ​mg	40 ​mg
115^a^	42	Female	L5	No	–	–
116	57	Female	L4	No	–	–

a Indicates responders to amitriptyline (>30% reduction in pain after 8 weeks).

**Table 2:** Pain Scores according to numerical rating score (NRS) and Doleur Neuropathique 4 (DN4) at baseline (pre-treatment), after selective nerve root block and after amitriptyline.

Study ID	NRS	DN4	NRS Post SNRB	NRS Post Amitriptyline	DN4 Post Ami	Stay on Medication	Reason for continued use/cessation
101^a^	9	8	0	5	8	Yes	Pain reduction and improved sleep
102^a^	6	4	1	3	4	Yes	Pain reduction and improved sleep
103	9	7	0	9	7	No	Not effective, dry mouth
104	4	6	0	–	–	No	Not effective, lost to follow up
105^a^	9	7	3	5	7	Yes	Pain reduction
106	10	5	5	10	5	No	Not effective
107^a^	7	4	0	3	3	Yes	Pain reduction
108^a^	6	8	3	3	4	Yes	Pain reduction
109	6	6	0	7	5	No	Dry mouth/Fatigue
110	6	5	1	5	5	Yes	Pain reduction
111^a^	7	6	1	1	3	Yes	Pain reduction and improved sleep
112	4	8	1	6	4	No	Not effective
113^a^	5	4	1	2	3	Yes	Pain reduction/Improved sleep
114^a^	7	8	4	4	4	Yes	Pain reduction
115^a^	8	6	1	1	5	Yes	Pain reduction/Improved sleep
116	6	4	1	7	4	No	Not effective

a Indicated responders to amitriptyline (>30% reduction in pain scores according to NRS). SNRB: selective nerve root block.

**Table 3:** Comparison of demographics and baseline (pre-treatment) neuropeptides between responders to amitriptyline (>30% reduction in pain) and non-responders (<30% reduction in pain).

	Responders	Non- Responders	p-value (Fisher’s exact test)
Age	53.33 ​± ​2.944	47.14 ​± ​4.595	0.337
NRS	7.11 ​± ​0.455	6.43 ​± ​0.87	0.385
DN4	6.22 ​± ​0.54	5.86 ​± ​0.51	0.72
Opioids			
Taking opioids	5	1	
Not taking opioids	4	6	0.145

Male	3	3	
Female	6	4	1

Neuropeptides	Mean pg/ml (SEM)	Mean pg/ml (SEM)	p-value (Mann-Whitney test)
Eotaxin-1	29.79 ​± ​7.48	18.97 ​± ​3.62	0.47
Eotaxin-3	4.51 ​± ​1.15	8.29 ​± ​1.95	0.16
IFN-γ	0.76 ​± ​0.35	0.34 ​± ​0.07	0.62
IL-10	0.14 ​± ​0.03	0.09 ​± ​0.02	0.31
IL-12/IL-23p40	5.66 ​± ​0.99	4.75 ​± ​0.7	0.51
IL-12p70	0.07 ​± ​0.04	0.05 ​± ​0.007	0.81
IL-13	2.93 ​± ​0.29	2.98 ​± ​0.34	0.89
IL-15	4.96 ​± ​0.72	4.77 ​± ​0.62	0.68
IL-16	10 ​± ​0.86	11.92 ​± ​1	0.35
IL-17 ​A	0.46 ​± ​0.12	0.36 ​± ​0.07	0.78
IL-1α	0.7 ​± ​0.49	0.39 ​± ​0.12	0.54
IL-1β	0.23 ​± ​0.02	0.26 ​± ​0.05	0.83
IL-4	0.06 ​± ​0.01	0.07 ​± ​0.01	0.58
IL-5	0.77 ​± ​0.09	0.68 ​± ​0.11	0.4
IL-6	1.3 ​± ​0.22	1.28 ​± ​0.27	0.91
IL-7	1.24 ​± ​0.14	1.12 ​± ​0.22	0.47
IL-8	15.78 ​± ​2.37	17.88 ​± ​6.1	0.75
IP-10	258 ​± ​40.3	209.8 ​± ​43.94	0.4
MCP-1	387 ​± ​46.83	423.9 ​± ​55.1	0.68
MCP-4	10.58 ​± ​2.43	8.93 ​± ​2.69	0.75
MDC	57.16 ​± ​10.4	44.78 ​± ​7.87	0.46
MIP-1α	8.38 ​± ​1.2	5.59 ​± ​0.65	0.14
MIP-1β	11.19 ​± ​2.26	14.03 ​± ​3.72	0.46
TARC	10.99 ​± ​1.07	8.55 ​± ​0.46	0.09
TNF-α	0.52 ​± ​0.05	0.5 ​± ​0.06	0.91
VEGF-A	3.62 ​± ​0.53	4.295 ​± ​1.17	0.99

### 3.2 Cytokines, chemokine and neurotrophin analysis following amitriptyline treatment

**Fig. 2:**
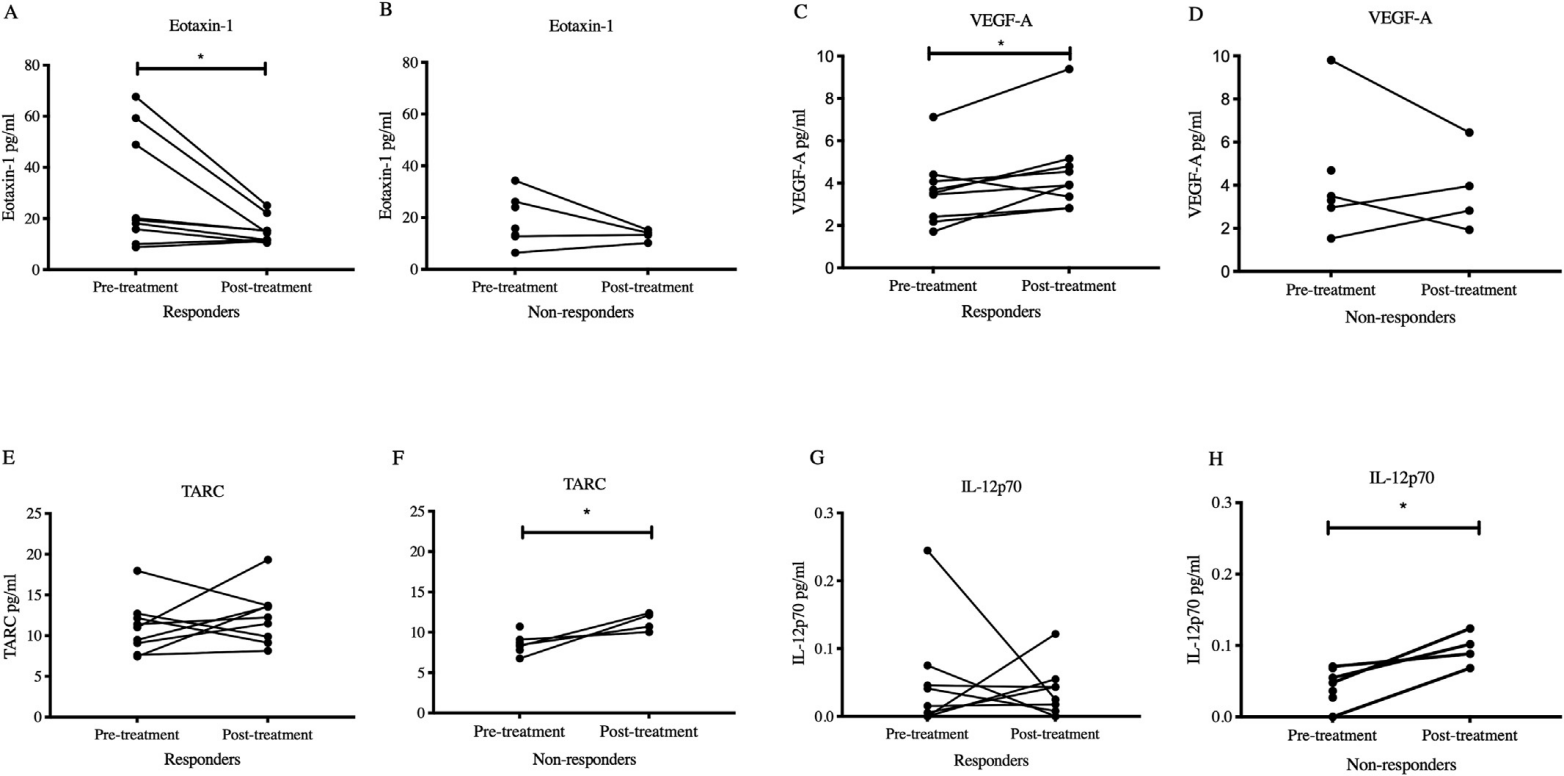
Eotaxin-1 (CCL11) is significantly reduced in the CSF of responders (n ​= ​9) after 8 weeks of amitriptyline [(Pre-treatment) 29.79 ​± ​7.48 ​pg/ml vs (Post-treatment) 15.26 ​± ​1.71 ​pg/ml, p ​= ​0.02] (2 ​A) but not in non-responders [(Pre-treatment) 18.97 ​± ​3.62 ​pg/ml vs (Post-treatment) 13.22 ​± ​1.07 ​pg/ml, p ​= ​0.52] (2 ​B). Vascular epidermal growth factor A (VEGF-A) is significantly increased in Cerebrospinal fluid (CSF) after eight weeks of amitriptyline in responders (>30% reduction in pain) [(Pre-treatment) 3.62 ​± ​0.53 ​pg/ml vs (Post-treatment) 4.45 ​± ​0.69, p ​= ​0.04] (2C), but not in non-responders [Pre-treatment 4.3 ​± ​1.18 ​pg/ml vs Post-treatment 3.79 ​± ​0.98 ​pg/ml, p ​= ​0.91] (2D). There is no significant difference in TARC (CCL17) in responders to amitriptyline [(Pre-treatment) 10.99 ​± ​1.07 ​pg/ml vs (Post-treatment) 12.35 ​± ​1.11 ​pg/ml, p ​= ​0.5] (2 ​E) but there is a significant increase in non-responders [(Pre-treatment, n ​= ​7) 8.55 ​± ​0.46 ​pg/ml vs (Post-treatment, n ​= ​4) 11.31 ​± ​0.57 ​pg/ml, p ​= ​0.02] (2 ​F). There is no significant difference in the concentration of IL-12p70 in responders to amitriptyline [(Pre-treatment) 0.05 ​± ​0.03 ​pg/ml vs (Post-treatment) 0.03 ​± ​0.01 ​pg/ml, p ​= ​0.7] (2G) but there is a significant increase in non-responders [(Pre-treatment, n ​= ​7) 0.05 ​± ​0.007 ​pg/ml vs (Post-treatment, n ​= ​4) 0.09 ​± ​0.01 ​pg/ml, p ​= ​0.03] (2H). Non-parametric paired (Wilcoxon Sign Rank) (2 ​A, C, E, G) and unpaired tests (Mann Whitney) (2 ​B, D, F, H) were used with values expressed in means with standard error of means (SEM).

There was a significant reduction in the chemokine eotaxin-1 (CCL11) in the post-treatment samples in comparison to the pre-treatment samples [(Pre-treatment) 29.79 ​± ​7.48 ​pg/ml vs (Post-treatment) 15.26 ​± ​1.71 ​pg/ml, p ​= ​0.02, n ​= ​9] in patient responders to amitriptyline (Fig. 2A, Table 4). There was no significant difference in eotaxin-1 in the non-responders between pre-treatment and post-treatment samples [(Pre-treatment) 18.97 ​± ​3.62 ​pg/ml vs (Post-treatment) 13.22 ​± ​1.07 ​pg/ml, p ​= ​0.52] (Fig. 2B, Table 4). Correlation analysis was performed to determine if levels of eotaxin-1 were related to pain scores and DN4 scores prior to treatment. There was no correlation identified with pain scores according to NRS (r ​= ​−0.233, CI -0.66 to 0.3116, p ​= ​0.38) and DN4 scores (r ​= ​0.27, CI -0.27 to 0.68, p ​= ​0.31) to eotaxin-1 levels pre-treatment. Correlation analysis was also performed to determine if there was a relationship between the percentage reduction in pain and change in eotaxin-1 (in the n ​= ​13 patients that had pre-treatment and post-treatment samples) after amitriptyline treatment. There was no correlation identified between these two variables (r ​= ​0.025, CI -0.5464 to 0.5804, p ​= ​0.93).

**Table 4:** Neuropeptide concentrations in pg/ml in Cerebrospinal fluid (CSF) before and after amitriptyline treatment for 8 weeks between responders (>30% reduction in pain) and non-responders (<30% reduction in pain).

	Responders			Non-Responders		
	Mean Pre-Drug (SEM) of neuropetides in pg/ml	Mean Post drug (SEM) of neuropetides in pg/ml	P value	Mean Pre-Drug (SEM) of neuropetides in pg/ml	Mean Post Drug (SEM) of neuropetides in pg/ml	P value
MCP-1	387 ​± ​46.82	390.5 ​± ​53.8	0.99	423.9 ​± ​55.1	423.7 ​± ​20.6	0.52
MCP-4	10.58 ​± ​2.43	8.85 ​± ​0.82	0.49	8.94 ​± ​2.7	11.38 ​± ​1.85	0.23
Eotaxin-3	4.51 ​± ​1.15	4.3 ​± ​1.22	0.99	8.29 ​± ​1.95	8.3 ​± ​3.88	0.88
Eotaxin-1	29.79 ​± ​7.48	15.26 ​± ​1.71	0.02∗	18.97 ​± ​3.62	13.22 ​± ​1.07	0.52
MIP-1α	8.38 ​± ​1.20	6.08 ​± ​0.6	0.1	5.6 ​± ​0.65	6.89 ​± ​0.75	0.32
MIP-1β	11.19 ​± ​2.26	9.25 ​± ​1.45	0.1	14.03 ​± ​3.72	11.31 ​± ​0.57	0.93
IP-10	258 ​± ​40.33	235 ​± ​15.34	0.65	209.8 ​± ​43.9	196.8 ​± ​37.4	0.99
MDC	57.16 ​± ​10.4	35.02 ​± ​3.06	0.1	44.78 ​± ​7.88	39.18 ​± ​4.84	0.65
TARC	10.99 ​± ​1.07	12.35 ​± ​1.11	0.5	8.55 ​± ​0.46	11.31 ​± ​0.57	0.02∗
Fractalkine	4.953 ​± ​1.15	7.182 ​± ​1.94	0.31	10.9 ​± ​2.2	6.43 ​± ​3.1	0.18
IFN-γ	0.38 ​± ​0.22	0.31 ​± ​0.14	0.93	0.35 ​± ​0.07	0.22 ​± ​0.03	0.3
IL-10	0.11 ​± ​0.03	0.18 ​± ​0.05	0.11	0.1 ​± ​0.02	0.11 ​± ​0.02	0.92
IL-12p70	0.05 ​± ​0.03	0.03 ​± ​0.01	0.70	0.05 ​± ​0.007	0.09 ​± ​0.01	0.03∗
IL-13	2.9 ​± ​0.29	3.1 ​± ​0.22	0.28	2.98 ​± ​0.34	2.80 ​± ​0.43	0.61
IL-1β	0.24 ​± ​0.02	0.31 ​± ​0.04	0.09	0.26 ​± ​0.05	0.23 ​± ​0.06	0.79
IL-4	0.07 ​± ​0.01	0.08 ​± ​0.01	0.25	0.07 ​± ​0.01	0.08 ​± ​0.03	0.75
IL-6	1.31 ​± ​0.22	1.5 ​± ​0.24	0.20	1.28 ​± ​0.28	1.24 ​± ​0.09	0.68
IL-8	15.78 ​± ​2.37	14.95 ​± ​2.59	0.65	17.8 ​± ​6.08	12.72 ​± ​2.44	0.78
TNF-α	0.53 ​± ​0.0)	0.52 ​± ​0.05	0.91	0.5 ​± ​0.06	0.54 ​± ​0.06	0.79
IL-12/IL-23p40	5.7 ​± ​0.99	5.22 ​± ​1.02	0.36	4.75 ​± ​0.7	4.70 ​± ​0.57	0.99
IL-15	4.96 ​± ​0.72	4.45 ​± ​0.49	0.16	4.80 ​± ​0.62	4.61 ​± ​0.72	0.92
IL-16	10 ​± ​0.86	10.55 ​± ​0.81	0.5	11.92 ​± ​1.02	13.05 ​± ​1.73	0.41
IL-17 ​A	0.36 ​± ​0.12	0.46 ​± ​0.15	0.46	0.36 ​± ​0.08	0.31 ​± ​0.11	0.6
IL-5	0.78 ​± ​0.1	0.81 ​± ​0.06	0.57	0.69 ​± ​0.11	0.78 ​± ​0.11	0.65
IL-7	1.24 ​± ​0.14	1.30 ​± ​0.19	0.3	1.12 ​± ​0.22	1.33 ​± ​0.39	0.78
VEGF-A	3.62 ​± ​0.53	4.45 ​± ​0.69	0.04∗	4.3 ​± ​1.18	3.79 ​± ​0.98	0.91

Significantly higher concentrations of Vascular Endothelial Growth Factor (VEGF-A) were observed in the CSF of responder’s post-treatment, compared to pre-treatment [(Pre-treatment) 3.62 ​± ​0.53 ​pg/ml vs (Post-treatment) 4.45 ​± ​0.69 ​pg/ml, p ​= ​0.04, n ​= ​9] (Fig. 2C, Table 4). There was no significant difference between samples of VEGF-A in the non-responders [Pre-treatment 4.3 ​± ​1.18 ​pg/ml vs Post-treatment 3.79 ​± ​0.98 ​pg/ml, p ​= ​0.91] (Fig. 2D, Table 4). Correlation analysis was performed to determine if levels of VEGF-A were related to pain scores and DN4 scores prior to treatment. There was no correlation between pain scores (r ​= ​−0.2598, CI -0.6902 to 0.3065, p ​= ​0.34) and DN4 scores (−0.0128, CI -0.53 to 0.5152, p ​= ​0.97) to VEGF-A in the pre-treatment samples. To determine if there was a relationship between VEGF-A and percentage reduction in pain, correlation analysis was performed in the n ​= ​13 patients with paired samples. No correlation was identified (r ​= ​−0.3019, CI -0.7397 to 0.315, p ​= ​0.31).

**Fig. 3:**
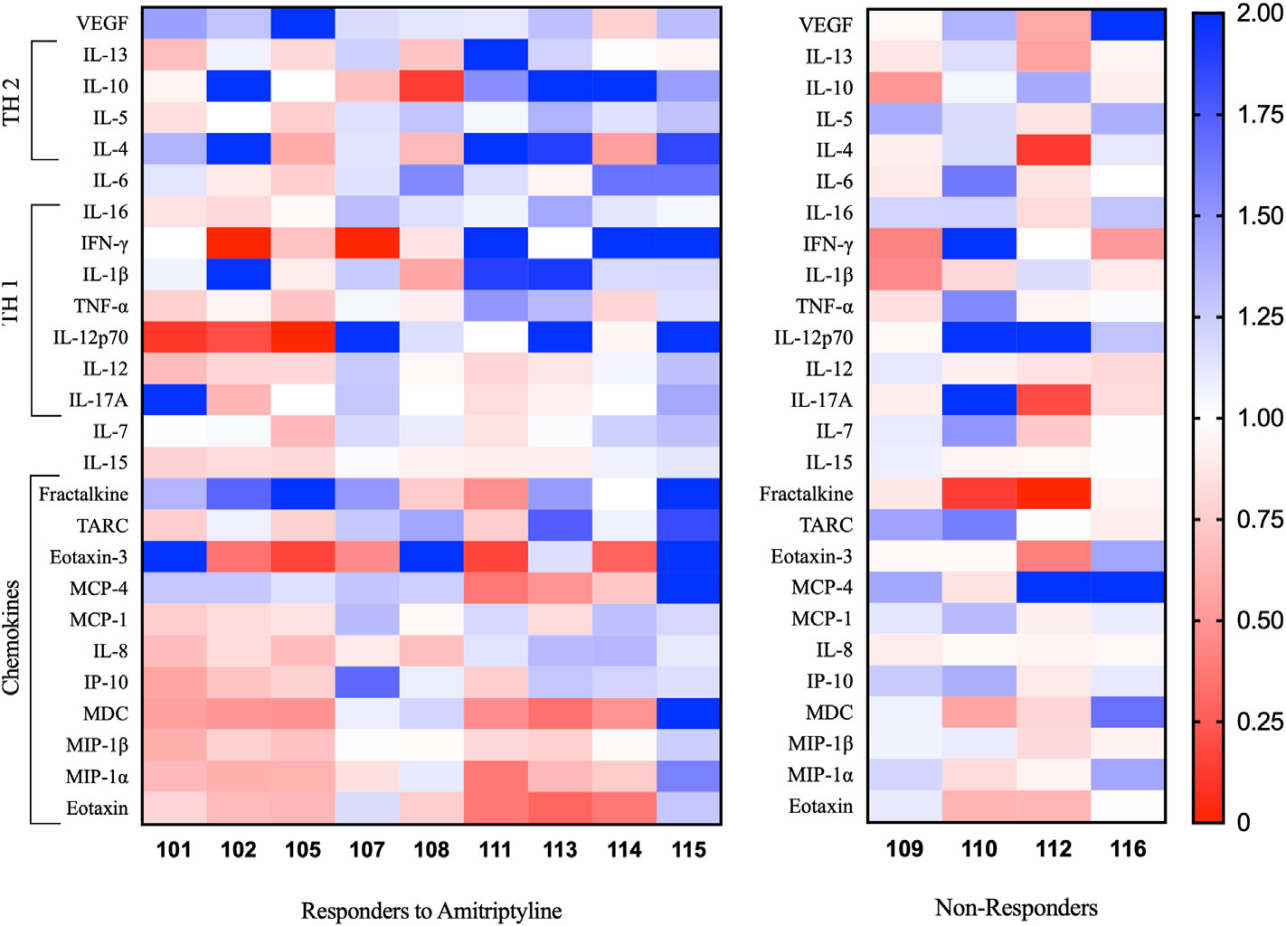
Heat maps of ratios between post-treatment samples (after 8 weeks of amitriptyline) in relation to baseline, pre-treatment samples in all patients with paired samples (N ​= ​9 in responders, N ​= ​4 in non-responders). The fractions are represented by colour according to range, blue being increased and red illustrating relevant decreases in concentration. Values of >2 are illustrated as if they were 2 (dark blue). (For interpretation of the references to colour in this figure legend, the reader is referred to the Web version of this article.)

There was no significant difference in TARC (CCL17) in responders to amitriptyline [(Pre-treatment) 10.99 ​± ​1.07 ​pg/ml vs (Post-treatment) 12.35 ​± ​1.11 ​pg/ml, p ​= ​0.5] (Fig. 2E, Table 4) but there was a significant increase in non-responders [(Pre-treatment, n ​= ​7) 8.55 ​± ​0.46 ​pg/ml vs (Post-treatment, n ​= ​4) 11.31 ​± ​0.57 ​pg/ml, p ​= ​0.02] (Fig. 2F, Table 4). There was no significant difference in the concentration of IL-12p70 in responders to amitriptyline [(Pre-treatment) 0.05 ​± ​0.03 ​pg/ml vs (Post-treatment) 0.03 ​± ​0.01 ​pg/ml, p ​= ​0.7] (Fig. 2G, Table 4) but there was a significant increase in non-responders [(Pre-treatment, n ​= ​7) 0.05 ​± ​0.007 ​pg/ml vs (Post-treatment, n ​= ​4) 0.09 ​± ​0.01 ​pg/ml, p ​= ​0.03] (Fig. 2H, Table 4). The results of the other neuropeptide changes in both groups are available in Table 4. GM-CSF, IL-2,TNF-β, NGF, GDNF and BDNF were undetectable in all samples. A link to the limits of detection and a reference are provided in section 2.6. To demonstrate the modulation of the individual neuropeptides in each individual patient, a heat map was created to illustrate the ratio of the post-treatment sample in comparison to the pre-treatment sample (Fig. 3). Although not significant in every case, there was a downward trend in chemokines within the responders compared to the non-responders.

### 3.3 Proteomics

**Fig. 4:**
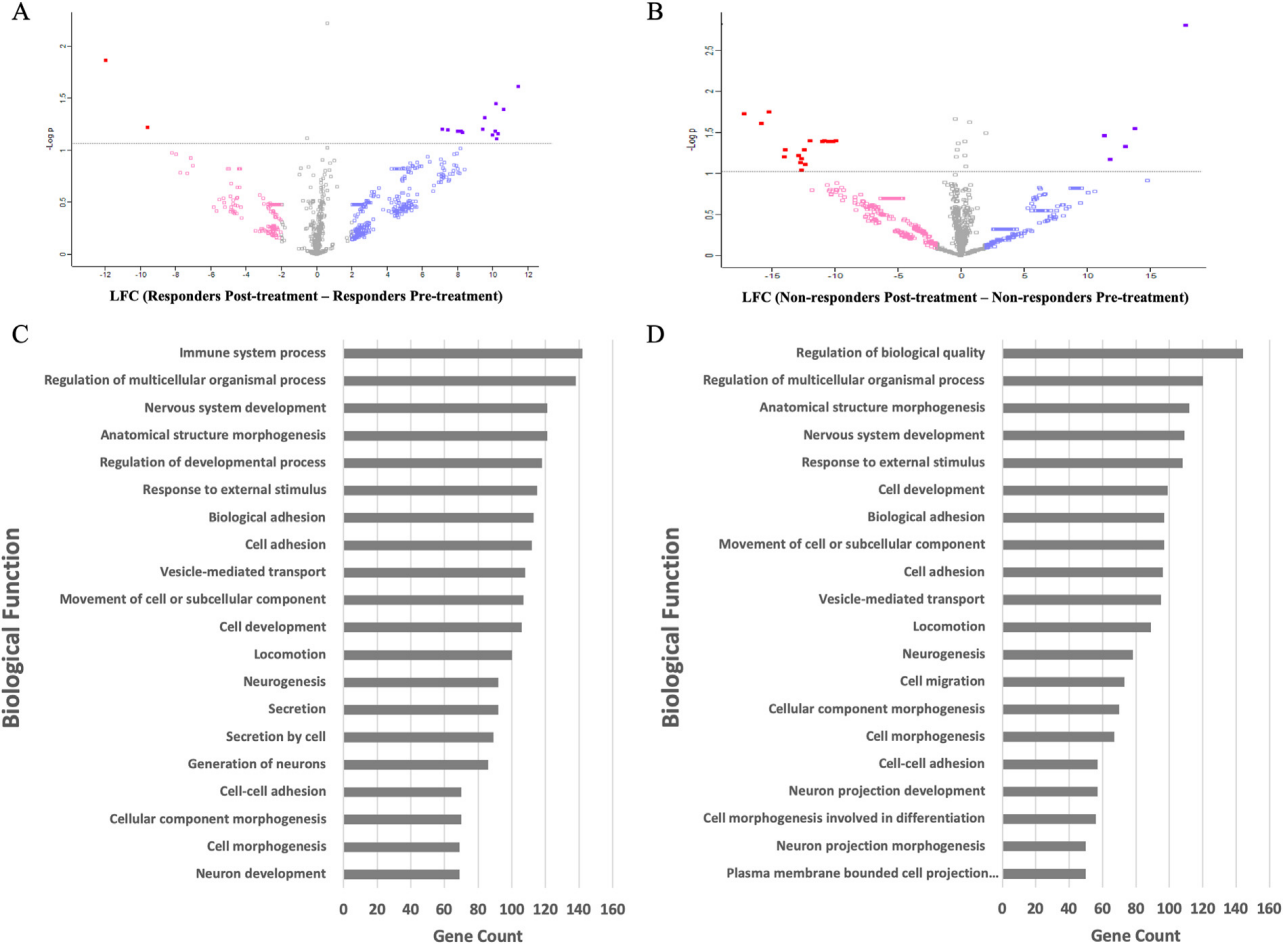
Volcano plots showing differential data of the 464 proteins differentially expressed in responders (4 ​A) to amitriptyline and the 416 differentially expressed proteins in non-responders (4 ​B). Red (decreased) and purple (increased) coloured squares indicate [-2 ​≤ ​Log Fold Change (LFC) ​≥ ​2] with False Discovery Rate (FDR) ​< ​0.05, using Log(p) ​> ​1.13 as a cut off for significantly altered proteins. Pink and blue hollow squares indicate (−2 ​≤ ​LFC ​≥ ​2) not significant by FDR. Grey dots are non-significant according to LFC. Proteins taken from Volcano plot with −2 ≤ LFC ≥2 were further analysed using Gene Otology (GO) analysis to show biological processes in responders (4C) and non-responders (4D). Bar charts illustrate the number of genes involved. (For interpretation of the references to colour in this figure legend, the reader is referred to the Web version of this article.)

CSF obtained from patients who responded positively to an 8-week course of amitriptyline resulted in the differential expression of 464 proteins compared to pre-treatment samples. Of these 464 differentially expressed proteins, 328 proteins were upregulated and 136 proteins were downregulated following amitriptyline treatment in responders (−2 < LFC >2) (Fig. 4A). The upregulated (Supplementary Table 1) and downregulated (Supplementary Table 2) proteins with LFC and FDR values in responders are available as supplementary material. Focusing on these differentially expressed proteins a total of 13 proteins were significantly upregulated (represented by Log(p) ​> ​1.13) (Table 5), while 2 proteins were significantly downregulated after amitriptyline (represented by Log(p) ​> ​1.13) (Table 6) (FDR<0.05). The upregulated proteins included Complement C1q tumor necrosis factor-related protein 5, Serine protease inhibitor Kazal-type 6, Tropomyosin alpha-4 chain, Immunoglobulin heavy variable 4–34, Titin, Inter-alpha-trypsin inhibitor heavy chain H3, Cadherin-11, Fibulin-7, Fetuin-B, Immunoglobulin heavy variable 1–18, Immunoglobulin lambda variable 3–16, Epithelial discoidin domain-containing receptor 1 and Semaphorin 6 ​A. The downregulated proteins were Aspartylglucosaminidase and Polypeptide N-acetylgalactosaminyltransferase 2.

**Table 5:** All significantly differentially up-regulated proteins in the responders cerebrospinal fluid (CSF) proteome post treatment according to Log fold change (LFC) > 2, False discover rate (FDR) ​< ​0.05 (represented by Log(p) ​> ​1.13) in order of LFC.

Proteins	Gene	LFC	LogP	FDR
Complement C1q tumor necrosis factor-related protein 5	C1QTNF5	11.42819	1.613784	0.046119
Serine protease inhibitor Kazal-type 6	SPINK6	10.61811	1.390578	0.041179
Tropomyosin alpha-4 chain	TPM4	10.31254	1.161122	0.016331
Immunoglobulin heavy variable 4-34	IGHV4-34	10.16426	1.44909	0.025403
Titin	TTN	10.15213	1.182481	0.003982
Inter-alpha-trypsin inhibitor heavy chain H3	ITIH3	9.997206	1.146764	0.001663
Cadherin-11	CDH11	9.563253	1.312062	0.035232
Fibulin-7	FBLN7	9.41027	1.199742	0.040171
Fetuin-B	FETUB	8.2742	1.171661	0.048387
Immunoglobulin heavy variable 1-18	IGHV1-18	8.185689	1.182731	0.005141
Immunoglobulin lambda variable 3-16	IGLV3-16	8.023568	1.184114	0.000655
Epithelial discoidin domain-containing receptor 1	DDR1	7.47055	1.193837	0.037399
Semaphorin 6 ​A	SEMA6A	7.141019	1.202043	0.002974

**Table 6:** All significantly differentially down-regulated proteins in the responders cerebrospinal fluid (CSF) proteome post treatment according to Log fold change (LFC) < −2, False discover rate (FDR) ​< ​0.05 (represented by Log(p) ​> ​1.13) in order of LFC.

Proteins	Gene	LFC	LogP	FDR
Aspartylglucosaminidase	AGA	−12.6602	1.771679	0.000403
Polypeptide N-acetylgalactosaminyltransferase 2	GALNT2	−9.60325	1.222043	0.000857

In comparison, CSF obtained from patients who did not respond to an 8-week course of amitriptyline (non-responders) resulted in the expression of 415 differentially expressed proteins (Figs. 4B), 185 proteins which were found to be upregulated and 230 proteins which were found to be downregulated (−2 < LFC >2). The upregulated (Supplementary Table 3) and downregulated (Supplementary Table 4) proteins with LFC and FDR values in the non-responders are available as supplementary material. Focusing on these differentially expressed proteins in non-responders, a total of 5 proteins were significantly upregulated (represented by Log(p) ​> ​1.13) (Table 7), while 20 proteins were significantly downregulated after amitriptyline (represented by Log(p) ​> ​1.13) (Table 8) (FDR<0.05).

**Table 7:** All significantly differentially up-regulated proteins in the non-responders cerebrospinal fluid (CSF) proteome post treatment according to Log fold change (LFC) ​≥ ​2, False discover rate (FDR) ​< ​0.05 (represented by Log(p) ​> ​1.13) in order of LFC.

Protein	Gene	LFC	LogP	FDR
V-type proton ATPase subunit S1	ATP6AP1	17.7330496	2.827164629	0.000714286
Phospholipase D4	PLD4	13.82809884	1.543076397	0.000761905
Polypeptide N-acetylgalactosaminyltransferase 2	GALNT2	13.05085897	1.331418633	0.001714286
Vitamin K-dependent protein Z	PROZ	11.81862341	1.173749182	0.00052381
Coagulation factor XIII B chain	F13B	11.35208103	1.458896657	0.00347619

**Table 8:** All significantly differentially down-regulated proteins in the non-responders cerebrospinal fluid (CSF) proteome post treatment according to Log fold change (LFC) ​≤ ​−2, False discover rate (FDR) ​< ​0.05 (represented by Log(p) ​> ​1.13) in order of LFC.

Protein	Gene	LFC	LogP	FDR
Double-stranded RNA-specific editase 1	ADARB1	−17.2087869	1.727415111	9.52381E-05
Heat shock cognate 71 ​kDa protein	HSPA8	−15.82209035	1.612765452	0.000142857
Mannan-binding lectin serine protease 1	MASP1	−15.26076494	1.74757113	4.7619E-05
Protein FAM19A5	TAFA5	−14.02971186	1.201375765	0.000428571
Serotransferrin	TF	−13.95079844	1.289981347	0.000285714
Contactin-4	CNTN4	−12.88501344	1.219143136	0.000380952
Netrin-G1	NTNG1	−12.74417686	1.130274283	0.000571429
Contactin-6	CNTN6	−12.64343766	1.179428872	0.00047619
Semaphorin-3G	SEMA3G	−12.44467851	1.288608643	0.001809524
Beta-actin-like protein 2	ACTBL2	−11.97152758	1.394164245	0.004809524
Ephrin type-A receptor 5	EPHA5	−11.02044582	1.393979428	0.004904762
alpha-1,2-Mannosidase	MAN1B1	−10.86874819	1.394104693	0.004857143
Myelin-associated glycoprotein	MAG	−10.55789995	1.393460684	0.004952381
Transmembrane glycoprotein NMB	GPNMB	−10.25154924	1.390688251	0.005142857
Spectrin alpha chain, erythrocytic 1	SPTA1	−10.132967	1.390104493	0.005190476
Adhesion G protein-coupled receptor B1	ADGRB1	−9.916713238	1.394168899	0.004761905

The top 20 GO analysis biological processes involving the differentially expressed proteins in the responders and non-responders are illustrated in Fig. 4. The top five biological processes identified in the responders according to gene count (GC) were: immune system process (GC ​= ​142), regulation of multicellular organismal process (GC ​= ​138), anatomical structure morphogenesis (GC ​= ​121), regulation of nervous system development (GC ​= ​121) and regulation of developmental process (GC ​= ​118) (Fig. 4A). The top five biological processes identified in the non-responders were: regulation of biological quality (GC ​= ​144), regulation of multicellular organismal process (GC ​= ​120), anatomical structure morphogenesis (GC ​= ​112), nervous system development (GC ​= ​109) and response to external stimulus (GC ​= ​108) (Fig. 4B). The clear differential between groups in relation to GO analysis was those proteins related to immune system process in responders but not in the non-responder group.

**Fig. 5:**
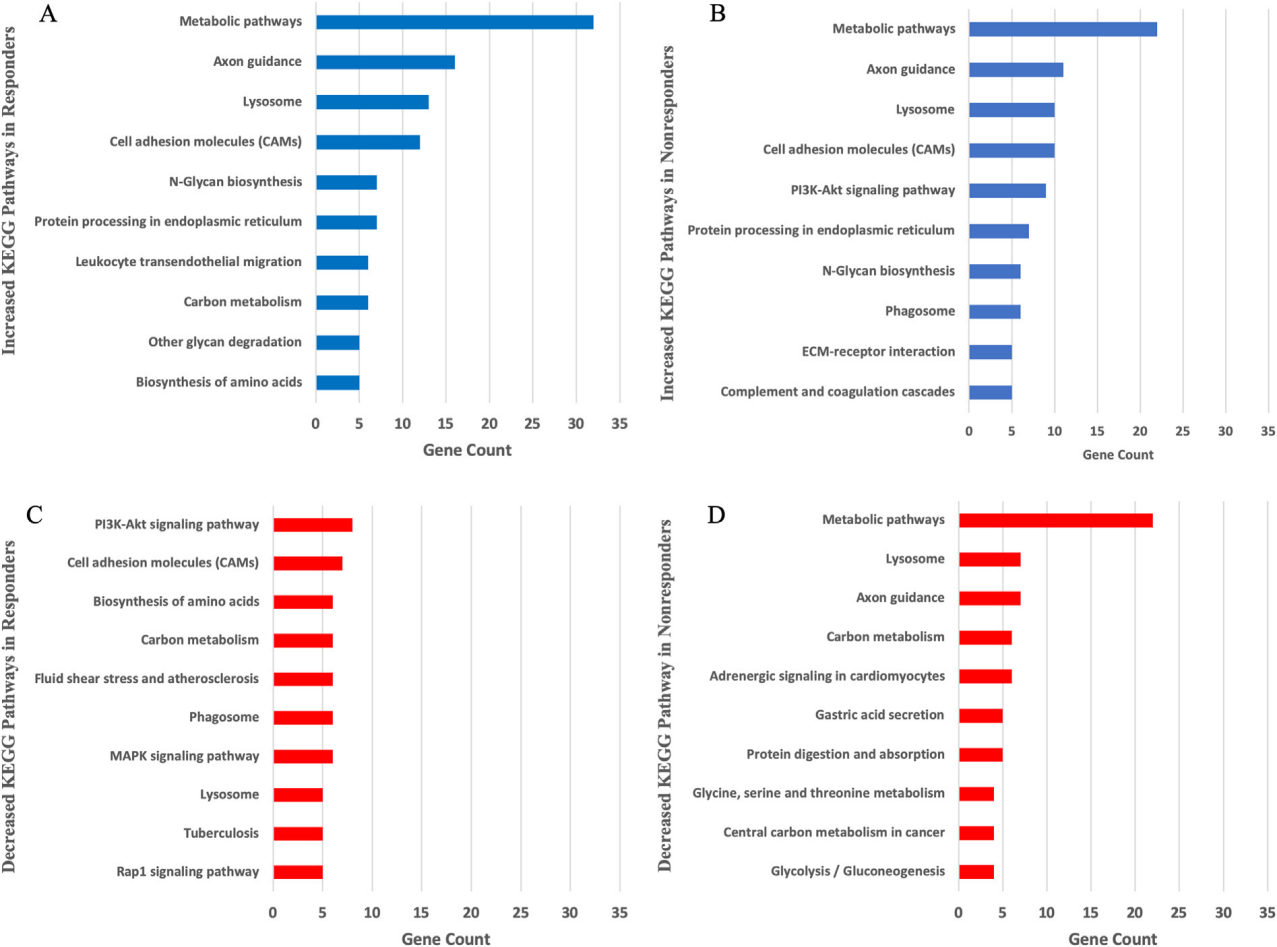
Proteins taken from Volcano plot with LFC ≥2 were further analysed using Kyoto Encyclopedia of Genes and Genomes (KEGG) pathway analysis in responders (5 ​A) and non-responders (5 ​B). Proteins taken from Volcano plot with LFC ​≤ ​−2 were analysed using KEGG pathway analysis in responders (5C) and non-responders (5D). Bar charts illustrate the number of genes involved.

KEGG analysis was subsequently preformed to identify the up and down regulated high-level functions of biological processes in the responders and non-responder group (Fig. 5). The most up regulated proteins according to KEGG analysis were those related to metabolic pathways in the responder (GC ​= ​32) and the non-responder (GC ​= ​22) groups (Fig. 5A and B). The second most upregulated proteins were related to axon guidance in the responders (GC ​= ​16) and non-responders (GC ​= ​11) (Fig. 5A and B). The most down regulated processes in responders according to GC were the PI3K-Akt signalling pathway (GC ​= ​8), cell adhesion molecules (CAMs) (GC ​= ​6) and mitogen-activated protein kinases (MAPK) signalling pathway (GC ​= ​6) (Fig. 5C). These pathways were not downregulated in the non-responders (Fig. 5D). The most downregulated processes in the non-responders were proteins related to metabolic pathways (GC ​= ​22), lysosome (GC ​= ​7) and axon guidance (GC ​= ​7) (Fig. 5D).

**Fig. 6:**
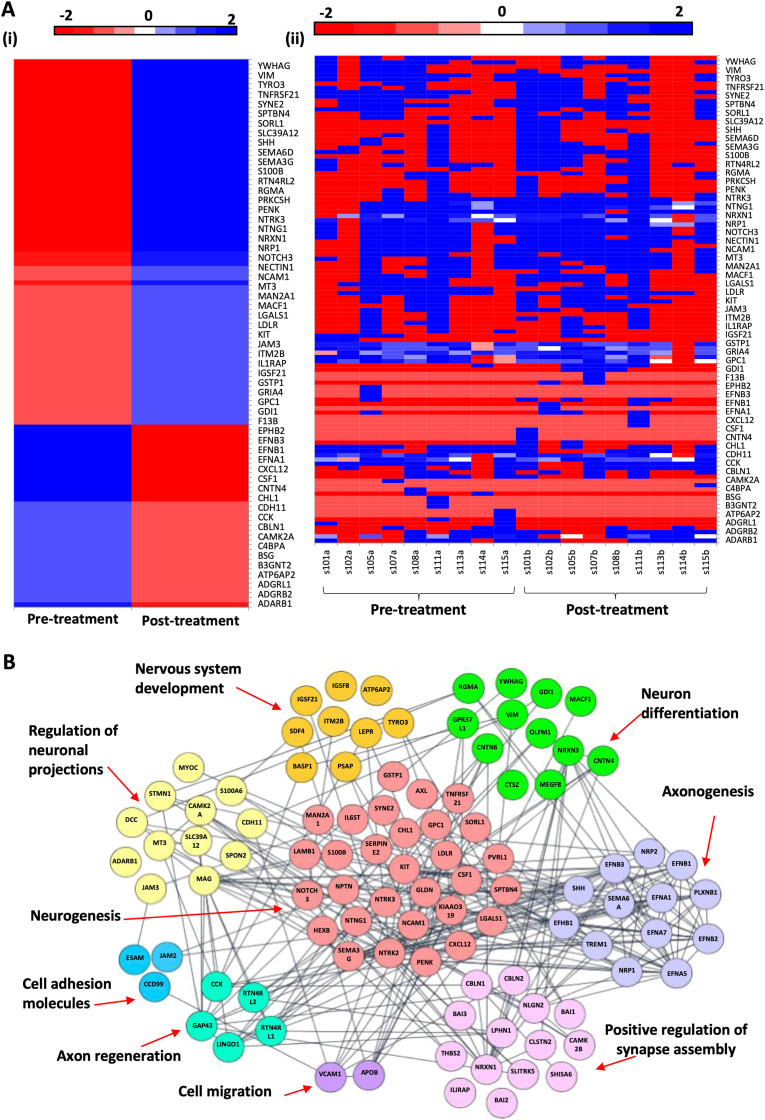
Proteins taken from Volcano plot with −2 ≤ LFC ≥2 were used to create heatmaps of the neural proteins in responders only. [6 ​A(i)] Panel shows the heatmap for all responder samples simply divided before (pre-treatment) and after 8 weeks of amitriptyline. [6 ​A (ii)] Panel breaks this heatmap down to illustrate each individual samples expression profile. (6 ​B) Clustering of the neuronal process protein network by biological function: Proteins taken from Volcano plot with −2 ​≤ ​LFC ​≥ ​2 were input into string where K-means clustering was performed to create this clustered network of biological processes.

Of the 464 differentially expressed proteins in the responders, the proteins were classified into protein classes as defined by the International Union of Basic and Clinical Pharmacology. Based on the modulation of proteins according to GO and KEGG pathways, we subdivided neuropeptides into neural proteins and immune process proteins to illustrate the dynamic changes of the relevant proteins under these two classes. The expression of neural proteins is illustrated in Fig. 6 with the up and down regulated proteins shown in a heat map, pre- and post-treatment in the responders (Fig. 6A). The relationship of these neural proteins is illustrated in a K-means clustered protein network of their interactions by biological function (Fig. 6B). The majority of proteins modulated were involved in neurogenesis, axonogenesis and regulation of neuronal projections and differentiation.

**Fig. 7:**
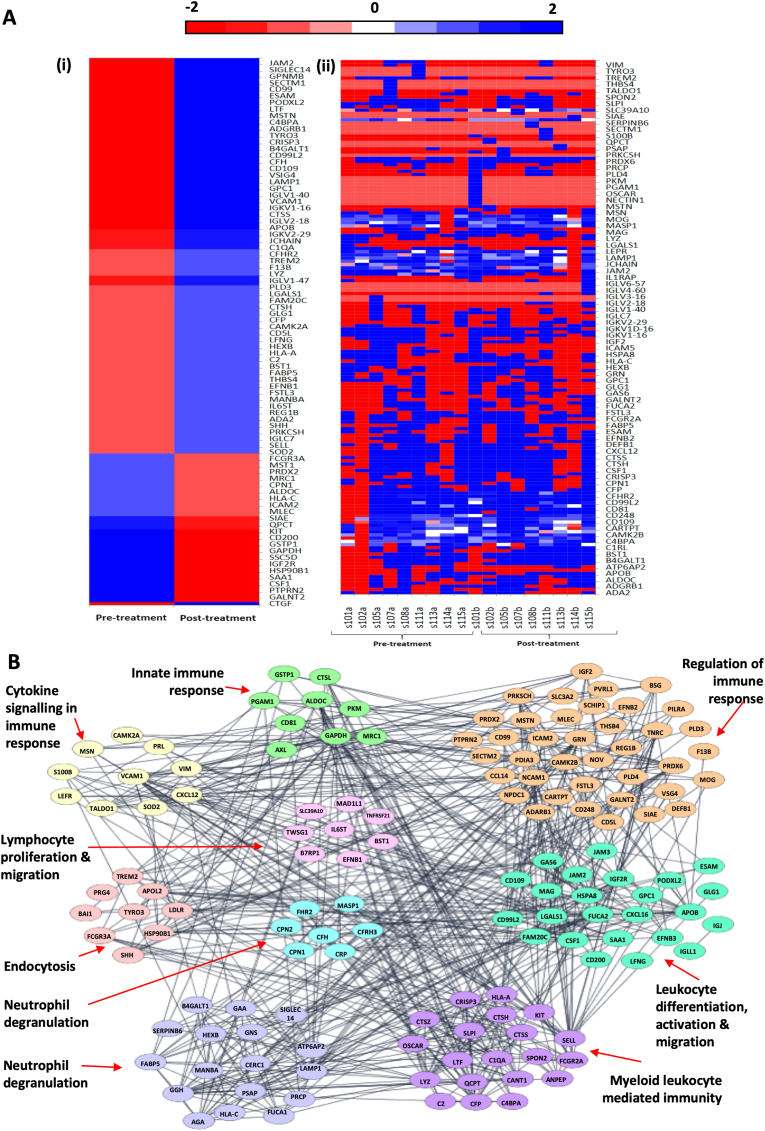
Proteins taken from Volcano plot with −2 ≤ LFC ≥2 were used to create heatmaps of the immune process proteins in responders only. [7 ​A(i)] Panel shows the heatmap for all responder samples simply divided before (pre-treatment) and after 8 weeks of amitriptyline. [7 ​A (ii)] Panel breaks this heatmap down to illustrate each individual samples expression profile. (7 ​B) Clustering of the immune related protein network by biological function: Proteins taken from Volcano plot with −2 ​≤ ​LFC ​≥ ​2 were input into string where K-means clustering was performed to create this clustered network according to biological processes.

Similarly, the expression of proteins related to immune processes are summarised in Fig. 7. The expression of immune proteins is illustrated with the up and down regulated proteins shown in a heat map, pre- and post-treatment in the responders (Fig. 7A). A clustered network of proteins also illustrated the largest concentrations of proteins according to biological function were related to regulation of immune response and leukocyte differentiation, activation and migration.

## 4 Discussion

We present the first *in vivo* study examining the effect of amitriptyline on the CSF secretome and proteome in patients treated with amitriptyline as therapy for CNP. Amitriptyline therapy resulted in 56% (9/16) of patients achieving a 30% reduction in pain which is concordant with other studies published (Finnerup et al., 2015; Gelijkens et al., 2014; Moore et al., 2015). A high response rate is due to deeming a >30% reduction as success, as opposed to 50% utilised in other selective studies (Finnerup et al., 2015; Moore et al., 2015). The reason why patients respond to tricyclic antidepressant medication likely relates to genetic polymorphisms (Brosen, 2004; Ryu et al., 2017) and to phenotypical characterisations of neuropathic pain (Baron et al., 2017). The choice to include patients in the non-responder group who had not provided a post treatment CSF sample was based on intention to treat.

The results from the proteomic GO and KEGG analysis illustrate many of the same active processes in both the responders and non-responders. For instance, nervous system development was one of the highest modulated processes according to GO analysis in both groups. KEGG analysis demonstrated that proteins related to axon guidance were the second most upregulated proteins after amitriptyline therapy in responders and non-responders. The neurotrophic effect of amitriptyline has been described, associated with increases of GDNF (Hisaoka et al., 2011; Hisaoka-Nakashima et al., 2015; Paumier et al., 2015), BDNF (Hisaoka-Nakashima et al., 2016; Paumier et al., 2015) and VEGF (Greene et al., 2009; Mackenzie and Ruhrberg, 2012; Ruiz de Almodovar et al., 2009; Warner-Schmidt and Duman, 2008). While we did not detect BDNF and GDNF within our samples, VEGF-A was significantly upregulated in responders suggesting a potential pathway of analgesic efficacy. While some of the processes listed in both groups may still be contributory to the analgesic effect of amitriptyline in neuropathic pain, the differences are likely to be more representative of this effect. Modulation of proteins related to immune system process were the most differentiated after amitriptyline in responders and did not feature in the top 20 processes of non-responders. This provides more compelling evidence that amitriptyline exerts its analgesic effect at least in part by immunomodulation. The immunomodulatory effects of amitriptyline have been described in microglia (Hisaoka-Nakashima et al., 2016; Hutchinson et al., 2010; Obuchowicz et al., 2006), astrocytes (Valera et al., 2014) and peripheral immune cells including T cells (Royds et al., 2020b) which can infiltrate the CNS after nerve injury and can be pathognomonic of neuropathic pain (Duffy et al., 2018). Although we did not observe statistical significance regarding modulation of cytokines from our data, definite trends were identified particularly a reduction in chemokines in responders.

The PI3K-Akt signalling pathway is implicated in many cellular processes including trafficking, immunity, proliferation and metabolism (Brunet et al., 2001; Xie et al., 2014), which we report here as the most downregulated pathway in responders. Specifically, there is *in vitro* evidence of PI3K inhibitors modulating the secretion of cytokines including IL-6 and TNF-α in LPS stimulated monocytes and macrophages (Xie et al., 2014). The PI3K-Akt signalling pathway has also been implicated in the development of neuropathic pain and hyperalgesia in sciatic nerve ligation models, diabetic neuropathy, bone cancer pain, spinal cord injury and inflammatory pain (Chen et al., 2017). Furthermore, inhibition of PI3K in the spinal cord prevented pain behaviours in mice induced by planter incision (Xu et al., 2014). Given the PI3K-Akt signalling pathway was upregulated in non-responders, this enhances the evidence that downregulation of this pathway is instrumental in the analgesic effect of amitriptyline for neuropathic pain.

Responders to amitriptyline had a significant decrease in the chemokine eotaxin-1 in CSF. While there was no healthy control arm in this study, raised eotaxin-1 levels in the CSF have already been demonstrated in patients with lumbar radicular pain compared to healthy controls (Backryd et al., 2017). Increased levels of eotaxin-1 in blood samples have also been reported in patients suffering from depression but larger studies have found no difference compared to controls (Teixeira et al., 2018). However, eotaxin-1 within CSF is raised compared to controls in other neurodegenerative diseases including Alzheimer’s, Huntington’s and multiple sclerosis (MS) (Huber et al., 2018). American football players with chronic post traumatic encephalopathy also had elevated levels of eotaxin-1 in the brain and CSF on autopsy (Cherry et al., 2017). Based on our inclusion and exclusion criteria we do not believe any of these conflicting variables were relevant in our patient cohort. Eotaxins are a subfamily of eosinophil chemokines which have been implicated in allergic inflammation, inflammatory bowel disease and asthma (Garcia-Zepeda et al., 1996; Huber et al., 2018; Mishra et al., 1999). Eosinophils are not prevalent within the CSF except for infection or the presence of blood; this suggests that eotaxin-1 is secreted by other cells within the CNS and likely carries out a different function (Fulkerson and Boaz, 2008). Chemokines in the CSF are thought to be produced primarily by glial cells and are referred to as glial-transmitters (Backryd et al., 2017; Gao and Ji, 2010; Grace et al., 2014; Huber et al., 2018; Miller et al., 2009; Turner et al., 2014; White et al., 2007). Within the central nervous system (CNS) neurons, oligodendrocytes and astrocytes express receptors for eotaxin-1, indicating it is a participant in neuronal-glial communication (Yang et al., 2007). Furthermore, network analysis of IL-1β/TNF-α stimulated human astrocytes *in vitro* resulted in secretomes of not only eotaxin-1, but PI3K and ERK 1/2 pathways which were all downregulated in responders within our study (Choi et al., 2014). Astrocytes and microglia’s production of eotaxin-1 in an inflammatory environment also leads to immune cell trafficking (Choi et al., 2014; Yang et al., 2007). Eotaxin-1 specifically recruits microglia and increases reactive oxygen species inducing excitotoxic neuronal cell death (Parajuli et al., 2015). From our cluster analysis of immune proteins, leukocyte differentiation, activation and migration were one of the highest modulated clusters after amitriptyline therapy in responders (Fig. 7B). This also suggests that amitriptyline may modulate the trafficking of immunocompetent cells within the CNS.

Pre-clinical and *in vitro* attenuation of glial inflammatory pathways with amitriptyline has been reported but not with eotaxin-1 directly (Hutchinson et al., 2010; Obuchowicz et al., 2006; Tai et al., 2006; Valera et al., 2014). However, to our knowledge there are no studies examining the effect of amitriptyline on chemokines within the CNS thus far. Reactive glial cells have been illustrated in patients with lumbar radicular pain using radiolabelled translocator protein (TSPO), (a marker of gliosis), compared to controls in the dorsal horn and neuroforamina (Albrecht et al., 2018). TSPO is a more specific marker for microglia and astrocytes (Rupprecht et al., 2010). From our data in responders, proteins related to immune system processes were modulated to the greatest extent and this was also associated with a decrease in proteins related to MAPK signalling pathways. Semaphorin 6 ​A, a significantly upregulated protein, negatively regulates the ERK1 and ERK2 cascade which are part of the MAPK signalling pathway and have been associated with pain hypersensitivity (Ji et al., 2014). ERK is upregulated in neurons, microglia and astrocytes after neuronal injury in rodent models and may be implicated in the pathogenesis of neuropathic pain (Zhuang et al., 2005). Amitriptyline has been shown to inhibit both the ERK and MAPK pathways in neuropathic pain models in rodents (Kim et al., 2019). Our data adds to the available evidence that amitriptyline attenuates pro-inflammatory pathways within the CNS. Pre-clinical studies also indicate that these are established pathways relating to pathological pain within the neuroimmune interface (Grace et al., 2014).

Levels of VEGF-A increased in responders to amitriptyline after 8 weeks of treatment. Without a control arm we cannot compare baseline (pre-treatment) levels to normal subjects. However, patients with Failed Back Surgery Syndrome (FBSS), who have a similar pain distribution, have lower levels of VEGF in CSF compared to healthy controls (McCarthy et al., 2013). There is also evidence of a reduction in VEGF levels within CSF in patients suffering from stress, depression and after a suicide attempt (Isung et al., 2012a, 2012b; Nowacka and Obuchowicz, 2012), which are synonymous with the CNP experience. Depletion of VEGF can lead to dysfunction of the nervous system (Hohman et al., 2015; Mackenzie and Ruhrberg, 2012; Ruiz de Almodovar et al., 2009; Segi-Nishida et al., 2008; Storkebaum et al., 2004) and injection of VEGF into the spinal cord of rats has demonstrated activation of neural stem cells after spinal cord injury (Liu et al., 2019). The significant increase in VEGF-A suggests amitriptyline may induce restorative repair mechanisms as a consequence of nerve dysfunction in chronic neuropathic pain.

VEGF expression after the application of amitriptyline has been uniquely demonstrated in animal models in the hippocampus (Greene et al., 2009; Warner-Schmidt and Duman, 2007, 2008). RNA sequencing analysis also indicates VEGF-A is produced predominately by astrocytes and microglia and also by neurons, oligodendrocytes and endothelial cells (Zhang et al., 2016). The role of VEGF-A within the CNS involves neurogenesis, axon outgrowth, neuronal migration, gliogenesis and glia survival (Mackenzie and Ruhrberg, 2012; Ruiz de Almodovar et al., 2009). Outside of the CNS, VEGF predominant role is in angiogenesis. However, VEGF has demonstrated an improvement in nerve blood flow in models of diabetic and peripheral neuropathies (Ruiz de Almodovar et al., 2009; Schratzberger et al., 2001). Sensory neuropathy also improves with intramuscular injection of plasmid DNA encoding VEGF in diabetic patients (Simovic et al., 2001). Further evidence to elicit mechanisms outside of angiogenesis include intramuscular VEGF gene transfer improving sensory deficits without angiogenesis in the sciatic nerve of mice suggesting a different mechanism in neurons (Murakami et al., 2006). Other potential mechanisms of VEGF include neuroprotective effects in DRG cell bodies which have multiple receptors for VEGF (Mackenzie and Ruhrberg, 2012; Nowacka and Obuchowicz, 2012; Ruiz de Almodovar et al., 2009). There is pre-clinical evidence that amitriptyline, likely via TrkA phosphorylation, regenerated DRG neurons in a dose dependent manner in rodents (Zheng et al., 2016). This provides further evidence that amitriptyline can enhance neuronal growth and redevelopment. Pathological nerve damage is synonymous with lumbar radicular pain and neuropathic pain (Colloca et al., 2017; Ohtori et al., 2011), and amitriptyline’s function may partially reverse this process.

The increase in concentration of TARC and IL-12 in the non-responders may be explained by severity or progression of pathology. Both neuropeptides have been associated with an increase in neurodegeneration and neuroinflammation within the CSF of patients with MS (Narikawa et al., 2005; Nicoletti et al., 1996). Furthermore, attenuation of the GM-CSF/TARC pathway is under investigation as a potentially novel analgesic for osteoarthritis (Conaghan et al., 2019).

While this study offers valuable insights into a vastly understudied area, there are limitations which include a relatively small number of participants and a confounding variable of opioid medications in some of the patients. For this reason, the results of this study, although informative, should be taken as preliminary evidence in humans. Although CSF analysis of patients medicated with opioids correlated level of pain to levels of IL-6 and IL-10, these cytokines were not significantly altered in our cohort (Zin et al., 2010). A study of CSF in patients with CRPS demonstrated no difference in the level of cytokines with patients on or not on opioids (Alexander et al., 2005). We still do not have sufficient evidence to determine how opioids effect cytokines, chemokines and the proteomic constituents in CSF. There is however some *in vitro* data to suggest amitriptyline may restore the analgesic effect of opioids by inhibiting Toll like receptor (TLR)-2 & −4 signalling (Hutchinson et al., 2010).

The reduction in pain in responders with the associated change in neuropeptides may not be attributable to amitriptyline alone. Improved sleep was also reported by 5/9 (56%) of responders which may be a confounding variable. Quality of sleep has been reported as a potential confounding variable in other studies examining neuropeptides and cytokines in chronic pain patients (Backryd et al., 2017). The impact of sleep on neuropeptides as an independent variable is yet to be defined however. There are also many limitations to CSF analysis that are discussed in other publications (Aasebo et al., 2014; Lind et al., 2016; Royds et al., 2020a). These include blood contamination (Aasebo et al., 2014), rostral-caudal gradient of protein concentrations (Aasebo et al., 2014) and inability to detect specific neuropeptides implicated in CNP (Lind et al., 2016). There is also the confounding variable of differentially expressed proteins that have a high individual variance between samples (Hu et al., 2005), however, none of these proteins were significantly altered in our cohort.

## 5 Conclusion

In summary, we have demonstrated the dynamic modulation of the proteomic and neuropeptide constituents of CSF *in vivo* in patients medicated with amitriptyline for the treatment of CNP. The predominant differential pathways affected by amitriptyline related to immune activity with a reduction of neural-glial pro-inflammatory pathways and a neurotrophic effect. These findings support pre-clinical and *in vitro* work with amitriptyline which demonstrated pharmacodynamic changes within inflammatory and VEGF pathways in particular. This provides information regarding the mechanism of action of amitriptyline *in vivo* in humans and also insights into the pathophysiology of CNP.

## Funding

The study was funded by a research grant from the College of Anaesthesiologists of Ireland (Research Grant, 2016). The funding source had no role in design; collection, analysis and interpretation of data; in the writing of the article and the decision to submit the article for publication.

## Declaration of competing interest

The authors have no conflict of interest to declare.
