# Supplementary material for: Examination and characterisation of the effect of amitriptyline therapy for chronic neuropathic pain on neuropeptide and proteomic constituents of human cerebrospinal fluid
Source: Brain Behav Immun Health. 2020 Dec 7;10:100184. doi: 10.1016/j.bbih.2020.100184 (PMC8474617; doi:10.1016/j.bbih.2020.100184)
Supplement: Multimedia component 1 [file mmc1.docx]

**Supplement Table 1:** All differentially up-regulated proteins in the responders CSF proteome post treatment (Log fold change (LFC) >2) in order of log fold change

| **Proteins** | **Gene** | **LFC** | **LogP** | **FDR** |
| --- | --- | --- | --- | --- |
| Complement C1q tumor necrosis factor-related protein 5 | C1QTNF5 | 11.42819 | 1.613784 | 0.046119 |
| Serine protease inhibitor Kazal-type 6 | SPINK6 | 10.61811 | 1.390578 | 0.041179 |
| Tropomyosin alpha-4 chain | TPM4 | 10.31254 | 1.161122 | 0.016331 |
| Immunoglobulin lambda variable 3-9 | IGLV3-9 | 10.21451 | 1.106922 | 0.000706 |
| Immunoglobulin heavy variable 4-34 | IGHV4-34 | 10.16426 | 1.44909 | 0.025403 |
| Titin | TTN | 10.15213 | 1.182481 | 0.003982 |
| Inter-alpha-trypsin inhibitor heavy chain H3 | ITIH3 | 9.997206 | 1.146764 | 0.001663 |
| Cadherin-11 | CDH11 | 9.563253 | 1.312062 | 0.035232 |
| Fibulin-7 | FBLN7 | 9.41027 | 1.199742 | 0.040171 |
| Immunoglobulin kappa variable 2-30 | IGKV2-30 | 8.406116 | 0.815816 | 0.025302 |
| Fetuin-B | FETUB | 8.2742 | 1.171661 | 0.048387 |
| Immunoglobulin heavy variable 1-18 | IGHV1-18 | 8.185689 | 1.182731 | 0.005141 |
| Rab GDP dissociation inhibitor alpha | GDI1 | 8.181318 | 1.0182 | 0.032913 |
| Thymosin beta-4 | TMSB4X | 8.114562 | 0.77565 | 0.035837 |
| Inter-alpha-trypsin inhibitor heavy chain H4 | ITIH4 | 8.056394 | 0.783139 | 0.015171 |
| Immunoglobulin lambda variable 3-16 | IGLV3-16 | 8.023568 | 1.184114 | 0.000655 |
| Serotransferrin | TF | 7.993902 | 0.819508 | 0.01003 |
| Spectrin beta chain | SPTBN4 | 7.960633 | 0.969096 | 0.010131 |
| Neutral alpha-glucosidase AB | GANAB | 7.907829 | 0.852551 | 0.038861 |
| Ephrin type-A receptor 7 | EPHA7 | 7.823435 | 0.962394 | 0.039163 |
| Immunoglobulin lambda variable 1-40 | IGLV1-40 | 7.805276 | 0.8565 | 0.021371 |
| Cleavage stimulation factor subunit 3 | CSTF3 | 7.760961 | 0.800228 | 0.037903 |
| Gamma-enolase | ENO2 | 7.748377 | 0.770616 | 0.027016 |
| Vascular cell adhesion protein 1 | VCAM1 | 7.557629 | 0.769627 | 0.030544 |
| Plasma alpha-L-fucosidase | FUCA2 | 7.541192 | 0.716678 | 0.045867 |
| Immunoglobulin kappa variable 1-16 | IGKV1-16 | 7.477572 | 0.704933 | 0.024446 |
| Epithelial discoidin domain-containing receptor 1 | DDR1 | 7.47055 | 1.193837 | 0.037399 |
| Serum albumin | ALB | 7.379386 | 0.738568 | 0.004788 |
| N-acetyllactosaminide beta-1,3-N-acetylglucosaminyltransferase 2 | B3GNT2 | 7.254544 | 0.806971 | 0.04748 |
| Protein MENT | MENT | 7.238085 | 0.88352 | 0.045968 |
| Glia-derived nexin | SERPINE2 | 7.221541 | 0.753334 | 0.025806 |
| Heat shock 70 kDa protein 13 | HSPA13 | 7.195482 | 0.744521 | 0.034476 |
| Beta-defensin 1 | DEFB1 | 7.175533 | 0.768265 | 0.035383 |
| Golgi integral membrane protein 4 | GOLIM4 | 7.172052 | 0.826421 | 0.012903 |
| Cathepsin S | CTSS | 7.157917 | 0.738508 | 0.031956 |
| Sema domain, transmembrane domain (TM), and cytoplasmic domain, (Semaphorin) 6A, isoform CRA_d (Semaphorin-6A) | SEMA6A | 7.141019 | 1.202043 | 0.002974 |
| Protein disulfide-isomerase A3 | PDIA3 | 7.10535 | 0.755627 | 0.032813 |
| Laminin subunit beta-1 | LAMB1 | 7.100268 | 0.774906 | 0.013609 |
| Netrin receptor DCC | DCC | 7.094054 | 0.863468 | 0.011038 |
| Beta-hexosaminidase | HEXA | 7.078698 | 0.720209 | 0.014768 |
| Netrin-G1 | NTNG1 | 7.054076 | 0.693102 | 0.049647 |
| PITH domain-containing protein 1 | PITHD1 | 7.050322 | 0.910988 | 0.04622 |
| Leucine-rich repeat and immunoglobulin-like domain-containing nogo receptor-interacting protein 1 | LINGO1 | 6.951312 | 0.695356 | 0.044456 |
| Immunoglobulin lambda variable 2-18 | IGLV2-18 | 6.940876 | 0.766607 | 0.000605 |
| Twisted gastrulation protein homolog 1 | TWSG1 | 6.656445 | 0.661405 | 0.016079 |
| Apolipoprotein B-100 | APOB | 6.639441 | 0.603951 | 0.024093 |
| Dickkopf-related protein 3 | DKK3 | 6.426616 | 0.886635 | 0.048085 |
| Scavenger receptor cysteine-rich type 1 protein M130 | CD163 | 6.310534 | 0.936823 | 0.01255 |
| Immunoglobulin heavy variable 3-9 | IGHV3-9 | 5.9589 | 0.848535 | 0.021875 |
| Matrix Gla protein | MGP | 5.83186 | 0.843416 | 0.026663 |
| Hyaluronan-binding protein 2 | HABP2 | 5.728062 | 0.879887 | 0.03871 |
| CD99 antigen-like protein 2 | CD99L2 | 5.723296 | 0.574611 | 0.013861 |
| Haptoglobin-related protein | HPR | 5.695962 | 0.833086 | 0.019808 |
| Immunoglobulin lambda variable 8-61 | IGLV8-61 | 5.658897 | 0.452481 | 0.000454 |
| HCG2044074, isoform CRA_c | MIA-RAB4B | 5.645584 | 0.50891 | 0.039819 |
| Immunoglobulin kappa variable 1D-16 | IGKV1D-16 | 5.601301 | 0.467253 | 0.021018 |
| Latent-transforming growth factor beta-binding protein 4 | LTBP4 | 5.598522 | 0.827051 | 0.00499 |
| Complement factor H | CFH | 5.587201 | 0.455301 | 0.005746 |
| Cysteine-rich secretory protein 3 | CRISP3 | 5.468066 | 0.505741 | 0.015675 |
| Adhesion G protein-coupled receptor L1 | ADGRL1 | 5.456646 | 0.843628 | 0.018901 |
| Tyrosine-protein kinase receptor TYRO3 | TYRO3 | 5.440636 | 0.456398 | 0.014516 |
| Complement C1r subcomponent-like protein | C1RL | 5.421855 | 0.853968 | 0.047782 |
| Sex hormone-binding globulin, isoform CRA_a | SHBG | 5.403968 | 0.424022 | 0.015524 |
| Immunoglobulin kappa variable 2-24 | IGKV2-24 | 5.401406 | 0.479066 | 0.000907 |
| Immunoglobulin kappa variable 1-17 | IGKV1-17 | 5.35438 | 0.786853 | 0.020968 |
| Beta-1,4-galactosyltransferase 1 | B4GALT1 | 5.346389 | 0.468748 | 0.029637 |
| Parvalbumin alpha | PVALB | 5.320453 | 0.87417 | 0.009173 |
| Polypeptide N-acetylgalactosaminyltransferase 6 | GALNT6 | 5.287332 | 0.465829 | 0.043044 |
| Lysosome-associated membrane glycoprotein 1 | LAMP1 | 5.273044 | 0.631152 | 0.028427 |
| V-set and immunoglobulin domain-containing protein 4 | VSIG4 | 5.269673 | 0.517698 | 0.049546 |
| Immunoglobulin kappa variable 1D-39 | IGKV1D-39 | 5.266697 | 0.820211 | 0.020917 |
| VPS10 domain-containing receptor SorCS1 | SORCS1 | 5.251321 | 0.44398 | 0.043649 |
| Fibulin-2 | FBLN2 | 5.240287 | 0.796701 | 0.036442 |
| Neuron-specific vesicular protein calcyon | CALY | 5.234496 | 0.495995 | 0.047581 |
| Glypican-1 [Cleaved into: Secreted glypican-1] | GPC1 | 5.215464 | 0.442803 | 0.033216 |
| Stanniocalcin-2 | STC2 | 5.211575 | 0.569803 | 0.0187 |
| (Death receptor 6) (CD antigen CD358) | TNFRSF21 | 5.211296 | 0.788606 | 0.018397 |
| Immunoglobulin heavy variable 1-69 | IGHV1-69 | 5.210403 | 0.505993 | 0.021673 |
| Hypoxia up-regulated protein 1 | HYOU1 | 5.205248 | 0.832738 | 0.002218 |
| Immunoglobulin kappa variable 2-40 | IGKV2-40 | 5.185853 | 0.823654 | 0.001714 |
| Cell growth regulator with EF hand domain protein 1 | CGREF1 | 5.155449 | 0.448906 | 0.045363 |
| Laminin subunit gamma-1 | LAMC1 | 5.133603 | 0.455035 | 0.028327 |
| C-C motif chemokine 14 | CCL14 | 5.130085 | 0.484322 | 0.039768 |
| Immunoglobulin heavy variable 3/OR16-12 | IGHV3OR16-12 | 5.095563 | 0.409347 | 0.001008 |
| CD109 antigen | CD109 | 5.077099 | 0.471812 | 0.041381 |
| Tissue alpha-L-fucosidase | FUCA1 | 5.029775 | 0.424189 | 0.023992 |
| Insulin-like growth factor-binding protein 5 | IGFBP5 | 5.019242 | 0.729684 | 0.031855 |
| Semaphorin-3G | SEMA3G | 5.009993 | 0.481919 | 0.047228 |
| Ryanodine receptor 3 | RYR3 | 5.001 | 0.823592 | 0.002268 |
| Protein FAM69C | DIPK1C | 4.989687 | 0.411878 | #N/A |
| Out at first protein homolog | OAF | 4.98203 | 0.745472 | 0.041734 |
| Intercellular adhesion molecule 5 | ICAM5 | 4.967385 | 0.467737 | 0.049093 |
| UDP-glucose 4-epimerase | GALE | 4.950185 | 0.465081 | 0.040373 |
| Semaphorin-6D | SEMA6D | 4.940383 | 0.481175 | 0.043196 |
| Follistatin-related protein 5 | FSTL5 | 4.933141 | 0.432481 | 0.042843 |
| Collagen alpha-1 | COL14A1 | 4.920002 | 0.590701 | 0.037097 |
| Cadherin-6 | CDH6 | 4.919758 | 0.826578 | 0.010585 |
| Podocalyxin-like protein 2 | PODXL2 | 4.913977 | 0.422657 | 0.047681 |
| Pregnancy zone protein | PZP | 4.906181 | 0.478639 | 0.030746 |
| Endothelial cell-selective adhesion molecule | ESAM | 4.887591 | 0.438144 | 0.013105 |
| Thrombospondin-2 | THBS2 | 4.883135 | 0.44767 | 0.033266 |
| Carboxypeptidase N subunit 2 | CPN2 | 4.881493 | 0.707047 | 0.03125 |
| Folate receptor beta | FOLR2 | 4.861958 | 0.732126 | 0.01124 |
| Stromal cell-derived factor 1 | CXCL12 | 4.85428 | 0.590177 | 0.034325 |
| Repulsive guidance molecule A | RGMA | 4.85224 | 0.719941 | 0.003931 |
| Lactotransferrin | LTF | 4.846201 | 0.387258 | 0.010988 |
| Testican-2 | SPOCK2 | 4.845342 | 0.427862 | 0.043851 |
| SLIT and NTRK-like protein 5 | SLITRK5 | 4.82721 | 0.445689 | 0.019052 |
| Immunoglobulin superfamily member 21 | IGSF21 | 4.811787 | 0.44693 | 0.044708 |
| Growth/differentiation factor 8 | MSTN | 4.803275 | 0.82346 | 0.017238 |
| Cadherin-15 | CDH15 | 4.798098 | 0.432784 | 0.035333 |
| Prosaposin receptor GPR37L1 | GPR37L1 | 4.748311 | 0.460093 | 0.018196 |
| Antileukoproteinase | SLPI | 4.748151 | 0.416171 | 0.02379 |
| Neural cell adhesion molecule L1-like protein | CHL1 | 4.739815 | 0.436375 | 0.002419 |
| CD99 | CD99 | 4.696795 | 0.823597 | #N/A |
| Protocadherin alpha-C2 | PCDHAC2 | 4.690416 | 0.412488 | 0.049798 |
| Alpha-N-acetylgalactosaminide alpha-2,6-sialyltransferase 1 | ST6GALNAC1 | 4.683117 | 0.496356 | 0.047278 |
| Transaldolase | TALDO1 | 4.675637 | 0.548778 | 0.012349 |
| Vimentin variant 3 | VIM | 4.660543 | 0.823026 | 0.007863 |
| Secreted and transmembrane protein 1 | SECTM1 | 4.657811 | 0.515337 | 0.015927 |
| Protocadherin-10 | PCDH10 | 4.64554 | 0.822966 | 0.047984 |
| Cochlin | COCH | 4.632325 | 0.354767 | 0.013558 |
| Multiple epidermal growth factor-like domains protein 9 | MEGF9 | 4.621635 | 0.414851 | 0.04627 |
| Immunoglobulin heavy variable 3-43 | IGHV3-43 | 4.605075 | 0.390951 | 0.004335 |
| Neural proliferation differentiation and control protein 1 | NPDC1 | 4.604126 | 0.46663 | 0.040423 |
| Junctional adhesion molecule B | JAM2 | 4.583459 | 0.560519 | 0.014315 |
| Contactin-6 | CNTN6 | 4.574779 | 0.425 | 0.049395 |
| Polypeptide N-acetylgalactosaminyltransferase 16 | GALNT16 | 4.571223 | 0.823634 | 0.042792 |
| Xyloside xylosyltransferase 1 | XXYLT1 | 4.565662 | 0.405786 | 0.042893 |
| SH3 domain-binding glutamic acid-rich-like protein 3 | SH3BGRL3 | 4.564519 | 0.823694 | 0.040524 |
| Sialic acid-binding Ig-like lectin 14 | SIGLEC14 | 4.555713 | 0.374308 | 0.03755 |
| Beta-galactoside alpha-2,6-sialyltransferase 2 | ST6GAL2 | 4.545533 | 0.821203 | 0.044758 |
| Peroxiredoxin-6 | PRDX6 | 4.544462 | 0.823268 | 0.032661 |
| Protocadherin-1 | PCDH1 | 4.534196 | 0.413003 | 0.037349 |
| Dihydrolipoyl dehydrogenase | DLD | 4.521311 | 0.823063 | 0.011542 |
| Transmembrane glycoprotein NMB | GPNMB | 4.516885 | 0.500012 | 0.038911 |
| Dyslexia-associated protein KIAA0319 | KIAA0319 | 4.51463 | 0.823572 | 0.040675 |
| Neuropilin-1 | NRP1 | 4.471499 | 0.414869 | 0.011391 |
| Ephrin-B2 | EFNB2 | 4.465533 | 0.454502 | 0.034879 |
| Adhesion G protein-coupled receptor B2 | ADGRB2 | 4.449862 | 0.370146 | 0.007258 |
| Sia-alpha-2,3-Gal-beta-1,4-GlcNAc-R:alpha 2,8-sialyltransferase | ST8SIA3 | 4.444735 | 0.411258 | 0.017641 |
| Cerebellin-2 | CBLN2 | 4.439655 | 0.526334 | 0.015978 |
| Zona pellucida sperm-binding protein 2 | ZP2 | 4.436124 | 0.426916 | 0.037147 |
| 4F2 cell-surface antigen heavy chain | SLC3A2 | 4.431887 | 0.373736 | 0.0125 |
| Protein S100-A4 | S100A4 | 4.429987 | 0.823618 | 0.032056 |
| Adhesion G protein-coupled receptor B1 | ADGRB1 | 4.401764 | 0.823217 | 0.010786 |
| Mannosyl-oligosaccharide 1,2-alpha-mannosidase IC | MAN1C1 | 4.369495 | 0.433085 | 0.047077 |
| Neuroendocrine protein 7B2 | SCG5 | 4.258281 | 0.403444 | 0.024899 |
| Sodium channel subunit beta-3 | SCN3B | 4.229204 | 0.822942 | 0.012046 |
| Lysosomal alpha-glucosidase | GAA | 4.228729 | 0.49497 | 0.027823 |
| C4b-binding protein alpha chain | C4BPA | 4.116539 | 0.535826 | 0.023841 |
| Phosphoserine aminotransferase | PSAT1 | 4.094867 | 0.415361 | 0.049899 |
| Immunoglobulin lambda-like polypeptide 1 | IGLL1 | 3.773476 | 0.431085 | 0.029738 |
| Immunoglobulin lambda variable 3-19 | IGLV3-19 | 3.53469 | 0.636991 | 0.021472 |
| Immunoglobulin lambda variable 1-47 | IGLV1-47 | 3.501144 | 0.555627 | 0.02127 |
| Decorin | DCN | 3.274455 | 0.602985 | 0.026159 |
| Immunoglobulin J chain | JCHAIN | 3.244743 | 0.571762 | 0.020817 |
| Follistatin-related protein 1 | FSTL1 | 3.229605 | 0.558961 | 0.037802 |
| Desmocollin-2 | DSC2 | 3.208931 | 0.335507 | 0.036794 |
| Cholecystokinin | CCK | 3.206945 | 0.240078 | 0.025252 |
| Latent-transforming growth factor beta-binding protein 2 | LTBP2 | 3.133864 | 0.602743 | 0.013407 |
| Protein NOV homolog | NOV | 3.100965 | 0.560999 | 0.034576613 |
| Insulin-like growth factor II | IGF2 | 3.074413 | 0.499014 | 0.020766 |
| Reticulon-4 receptor-like 2 | RTN4RL2 | 2.99753 | 0.295915 | 0.041835 |
| Delta and Notch-like epidermal growth factor-related receptor | DNER | 2.995368 | 0.299798 | 0.043145 |
| Ig-like domain-containing protein | n/a | 2.981627 | 0.496303 | 0.039393489 |
| Immunoglobulin heavy variable 1-2 | IGHV1-2 | 2.978094 | 0.25598 | 0.031401 |
| Immunoglobulin kappa variable 2-29 | IGKV2-29 | 2.967857 | 0.215379 | 0.007308 |
| Immunoglobulin lambda variable 4-60 | IGLV4-60 | 2.957717 | 0.225099 | 0.000504 |
| Protocadherin Fat 2 | FAT2 | 2.940774 | 0.504804 | 0.04753 |
| Heparan-sulfate 6-O-sulfotransferase 3 | HS6ST3 | 2.902526 | 0.307931 | 0.04254 |
| Probable serine carboxypeptidase CPVL | CPVL | 2.892378 | 0.514199 | 0.046371 |
| Apolipoprotein M | APOM | 2.889507 | 0.281454 | 0.019153 |
| EGF-containing fibulin-like extracellular matrix protein 2 | EFEMP2 | 2.887198 | 0.511931 | 0.012298 |
| Complement C1q subcomponent subunit A | C1QA | 2.87699 | 0.26346 | 0.023034 |
| Nectin-1 | NECTIN1 | 2.875081 | 0.277922 | 0.039113 |
| ICOS ligand (Inducible T-cell co-stimulator ligand, isoform CRA_b) | ICOSLG | 2.846608 | 0.49537 | 0.016129 |
| Soluble calcium-activated nucleotidase 1 | CANT1 | 2.824137 | 0.288835 | 0.043548 |
| C-type lectin domain family 11 member A | CLEC11A | 2.822682 | 0.29778 | 0.049496 |
| Phosphoglycerate kinase 1 | PGK1 | 2.820843 | 0.249104 | 0.019607 |
| Complement factor H-related protein 2 | CFHR2 | 2.818446 | 0.51614 | 0.033518 |
| Kunitz-type protease inhibitor 2 | SPINT2 | 2.813099 | 0.509133 | 0.017692 |
| Triggering receptor expressed on myeloid cells 2 | TREM2 | 2.811125 | 0.206033 | 0.047732 |
| Sulfhydryl oxidase 2 | QSOX2 | 2.807902 | 0.219517 | 0.041482 |
| Myelin-oligodendrocyte glycoprotein | MOG | 2.793473 | 0.223971 | 0.005796 |
| Selenoprotein P | SELENOP | 2.784963 | 0.455277 | 0.006552 |
| Cathepsin Z | CTSZ | 2.767259 | 0.481482 | 0.048185 |
| Coagulation factor XIII B chain | F13B | 2.749824 | 0.289933 | 0.024849 |
| Protein CutA (Acetylcholinesterase-associated protein) | CUTA | 2.747664 | 0.200404 | 0.018246 |
| Nidogen-1 | NID1 | 2.737216 | 0.478418 | 0.029284 |
| Protein CASC4 | CASC4 | 2.724995 | 0.478607 | 0.014415323 |
| Mannosyl-oligosaccharide 1,2-alpha-mannosidase IA | MAN1A1 | 2.712764 | 0.257372 | 0.033115 |
| Lysozyme C | LYZ | 2.710925 | 0.458936 | 0.035585 |
| Golgi membrane protein 1 | GOLM1 | 2.69706 | 0.481671 | 0.042944 |
| Immunoglobulin heavy variable 2-70D | IGHV2-70D | 2.688471 | 0.168082 | 0.005494 |
| C1QTNF3-AMACR readthrough (NMD candidate) | C1QTNF3-AMACR | 2.686661 | 0.261925 | 0.011794 |
| Alpha-mannosidase 2 | MAN2A1 | 2.670932 | 0.198693 | 0.039869 |
| Immunoglobulin lambda variable 9-49 | IGLV9-49 | 2.658948 | 0.203054 | 0.004385 |
| L-selectin | SELL | 2.658451 | 0.26219 | 0.029183 |
| Protein shisa-7 | SHISA7 | 2.653551 | 0.200692 | 0.00751 |
| Lysosomal Pro-X carboxypeptidase | PRCP | 2.649717 | 0.450599 | 0.033921 |
| Superoxide dismutase | SOD2 | 2.637111 | 0.194333 | 0.0422179 |
| V-set and transmembrane domain-containing protein 2A | VSTM2A | 2.623182 | 0.40899 | 0.008871 |
| BDNF/NT-3 growth factors receptor | NTRK2 | 2.619208 | 0.460349 | 0.039718 |
| Immunoglobulin lambda constant 7 | IGLC7 | 2.61887 | 0.179362 | 0.007056 |
| Gliomedin [Cleaved into: Gliomedin shedded ectodomain] | GLDN | 2.615743 | 0.210857 | 0.041431 |
| Mitotic spindle assembly checkpoint protein MAD1 | MAD1L1 | 2.613108 | 0.478607 | 0.009829 |
| Neurexophilin-1 | NXPH1 | 2.610901 | 0.188477 | 0.009627 |
| Nesprin-2 | SYNE2 | 2.59432 | 0.478607 | 0.003175 |
| Protein-L-isoaspartate O-methyltransferase | PCMT1 | 2.591623 | 0.251897 | 0.003226 |
| Immunoglobulin lambda variable 6-57 | IGLV6-57 | 2.582448 | 0.190061 | 0.021623 |
| UPF0606 protein KIAA1549L | KIAA1549L | 2.563533 | 0.234911 | 0.014264 |
| Uncharacterized protein KIAA2026 | KIAA2026 | 2.560437 | 0.478607 | 0.040272 |
| A disintegrin and metalloproteinase with thrombospondin motifs 4 | ADAMTS4 | 2.556726 | 0.210405 | 0.018296 |
| Immunoglobulin kappa variable 1-27 | IGKV1-27 | 2.547243 | 0.196956 | 0.000958 |
| Low-density lipoprotein receptor | LDLR | 2.543712 | 0.235158 | 0.014466 |
| Inositol monophosphatase 3 | IMPAD1 | 2.543062 | 0.444115 | 0.047429435 |
| Polypeptide N-acetylgalactosaminyltransferase 1 | GALNT1 | 2.533654 | 0.280602 | 0.037702 |
| Growth hormone A1 | PRL | 2.528425 | 0.478607 | 0.020716 |
| Forkhead-associated domain-containing protein 1 | FHAD1 | 2.524438 | 0.478607 | 0.007964 |
| Follistatin-related protein 3 | FSTL3 | 2.523193 | 0.196615 | 0.019204 |
| Alpha-1,3-mannosyl-glycoprotein 2-beta-N-acetylglucosaminyltransferase | MGAT1 | 2.511858 | 0.209734 | 0.032107 |
| Interleukin-6 receptor subunit beta | IL6ST | 2.506559 | 0.408963 | 0.033669 |
| Apolipoprotein L1 | APOL1 | 2.504502 | 0.228614 | 0.017188 |
| Msx2-interacting protein | SPEN | 2.503723 | 0.478607 | 0.04506 |
| Granulins | GRN | 2.50017 | 0.478607 | 0.03246 |
| Eukaryotic translation initiation factor 2 subunit 3B | EIF2S3B | 2.4947 | 0.191611 | 0.03377 |
| Beta-mannosidase | MANBA | 2.494585 | 0.192063 | 0.016935 |
| Immunoglobulin heavy constant delta | IGHD | 2.483298 | 0.180974 | 0.003377 |
| Glucosidase 2 subunit beta | PRKCSH | 2.483261 | 0.426494 | 0.01623 |
| Immunoglobulin lambda variable 3-1 | IGLV3-1 | 2.479587 | 0.179397 | 0.021522 |
| Sonic hedgehog protein | SHH | 2.478774 | 0.478607 | 0.039214 |
| Junctional adhesion molecule C | JAM3 | 2.472368 | 0.208454 | 0.046069 |
| Osteoclast-associated immunoglobulin-like receptor | OSCAR | 2.467715 | 0.478607 | 0.001563 |
| Lithostathine-1-beta | REG1B | 2.467116 | 0.478607 | 0.02495 |
| Adenosine deaminase 2 | ADA2 | 2.466058 | 0.251314 | 0.00877 |
| Immunoglobulin kappa variable 6-21 | IGKV6-21 | 2.451476 | 0.205765 | 0.00504 |
| Immunoglobulin heavy variable 1-46 | IGHV1-46 | 2.449861 | 0.263652 | 0.021724 |
| Calsyntenin-2 | CLSTN2 | 2.444898 | 0.478607 | 0.046421 |
| Beta-1,3-N-acetylglucosaminyltransferase lunatic fringe | LFNG | 2.442935 | 0.478607 | 0.043095 |
| Noelin | OLFM1 | 2.437302 | 0.191334 | 0.045413 |
| Serglycin | SRGN | 2.435364 | 0.279198 | 0.027722 |
| Heat shock cognate 71 kDa protein | HSPA8 | 2.424019 | 0.205323 | 0.012147 |
| Carbohydrate sulfotransferase 10 | CHST10 | 2.419984 | 0.200331 | 0.017843 |
| Protein S100-B | S100B | 2.414356 | 0.478607 | 0.024345 |
| SPARC-related modular calcium-binding protein 1 | SMOC1 | 2.414143 | 0.234824 | 0.046472 |
| CD5 antigen-like | CD5L | 2.404987 | 0.174821 | 0.017944 |
| Transgelin | TAGLN | 2.401188 | 0.166881 | 0.036694 |
| Dyslexia-associated protein KIAA0319-like protein | KIAA0319L | 2.394599 | 0.178434 | 0.008165 |
| Desmocollin-3 | DSC3 | 2.393876 | 0.478607 | 0.03876 |
| HLA class I histocompatibility antigen, A-24 alpha chain | HLA-A | 2.39099 | 0.246376 | 0.025 |
| Immunoglobulin heavy variable 3-64 | IGHV3-64 | 2.389062 | 0.224358 | 0.000806 |
| Phospholipase D4 | PLD4 | 2.378785 | 0.175139 | 0.012601 |
| Beta-hexosaminidase subunit beta | HEXB | 2.37794 | 0.184693 | 0.02626 |
| Complement factor H-related protein 3 | CFHR3 | 2.375713 | 0.478607 | 0.036946 |
| Lactosylceramide 4-alpha-galactosyltransferase | A4GALT | 2.373926 | 0.478607 | 0.046976 |
| Neudesin | NENF | 2.365416 | 0.257174 | 0.049143 |
| Contactin-4 | CNTN4 | 2.364613 | 0.189003 | 0.042389 |
| Golgi apparatus protein 1, isoform CRA_c | GLG1 | 2.357638 | 0.478607 | 0.014617 |
| Immunoglobulin lambda variable 5-37 | IGLV5-37 | 2.356011 | 0.478607 | 0.000554 |
| Growth arrest-specific protein 6 | GAS6 | 2.347713 | 0.191653 | 0.038558 |
| Pro-cathepsin H | CTSH | 2.341582 | 0.384575 | 0.002319 |
| Chitinase-3-like protein 2 | CHI3L2 | 2.341572 | 0.478607 | 0.039365 |
| Group XV phospholipase A2 | PLA2G15 | 2.338137 | 0.478607 | 0.014718 |
| Neuroligin-2 | NLGN2 | 2.337719 | 0.193261 | 0.043246 |
| Matrix remodeling-associated protein 8 | MXRA8 | 2.33692 | 0.478607 | 0.045766 |
| Integrin beta-like protein 1 | ITGBL1 | 2.334031 | 0.199043 | 0.019254 |
| Extracellular serine/threonine protein kinase FAM20C | FAM20C | 2.331936 | 0.478607 | 0.04244 |
| Ephrin-B3 | EFNB3 | 2.327666 | 0.478607 | 0.039315 |
| Galectin-1 | LGALS1 | 2.325878 | 0.478607 | 0.027117 |
| Retbindin | RTBDN | 2.325822 | 0.478607 | 0.016179 |
| Piezo-type mechanosensitive ion channel component | PIEZO2 | 2.325282 | 0.243486 | 0.011341 |
| C-type mannose receptor 2 | MRC2 | 2.320588 | 0.179577 | 0.048034 |
| Phosphoglycerate mutase 1 | PGAM1 | 2.318655 | 0.478607 | 0.030393 |
| Protocadherin-9 | PCDH9 | 2.315718 | 0.173309 | 0.009123 |
| Protocadherin-17 | PCDH17 | 2.309916 | 0.239419 | 0.017288 |
| Phospholipase D3 | PLD3 | 2.30991 | 0.178863 | 0.042188 |
| Connective tissue growth factor | CTGF | 2.309777 | 0.478607 | 0.032560484 |
| Latent-transforming growth factor beta-binding protein 1 | LTBP1 | 2.285884 | 0.167376 | 0.009577 |
| Properdin | CFP | 2.282291 | 0.478607 | 0.032359 |
| Immunoglobulin heavy variable 2-26 | IGHV2-26 | 2.281675 | 0.213106 | 0.004234 |
| Neuropilin-2 | NRP2 | 2.281607 | 0.24503 | 0.000252 |
| Exostosin-like 2 | EXTL2 | 2.27606 | 0.205698 | 0.048135 |
| N-acetylglucosamine-6-sulfatase | GNS | 2.274445 | 0.478607 | 0.012651 |
| Cytokine-like protein 1 | CYTL1 | 2.260168 | 0.157572 | 0.047177 |
| Proteoglycan 4 | PRG4 | 2.259447 | 0.362453 | 0.0063 |
| Plexin-B1 | PLXNB1 | 2.257359 | 0.478607 | 0.017591 |
| Calcium/calmodulin-dependent protein kinase type II subunit alpha | CAMK2A | 2.255317 | 0.189784 | 0.049446 |
| EPHB2 protein (Ephrin type-B receptor 2) | EPHB2 | 2.254328 | 0.478607 | 0.008014 |
| Thioredoxin domain-containing protein 17 | TXNDC17 | 2.251837 | 0.478607 | 0.045716 |
| Plexin domain-containing protein 1 | PLXDC1 | 2.242517 | 0.163638 | 0.042087 |
| Voltage-dependent calcium channel subunit alpha-2/delta-2 | CACNA2D2 | 2.242261 | 0.478607 | 0.009677 |
| C-X-C motif chemokine 16 | CXCL16 | 2.230197 | 0.218445 | 0.046321 |
| Protocadherin gamma-C5 | PCDHGC5 | 2.225271 | 0.223459 | 0.049748 |
| Low-density lipoprotein receptor-related protein 11 | LRP11 | 2.224691 | 0.478607 | 0.040726 |
| Ceroid-lipofuscinosis neuronal protein 5 | CLN5 | 2.22155 | 0.171885 | 0.000302 |
| Protein FAM198B | FAM198B | 2.214528 | 0.478607 | 0.041129032 |
| Thrombospondin-4 | THBS4 | 2.212717 | 0.178051 | 0.011089 |
| Insulin-like growth factor binding protein 3 isoform b | IGFBP3 | 2.205069 | 0.161065 | 0.007661 |
| Zinc transporter ZIP10 | SLC39A10 | 2.204511 | 0.158624 | 0.048942 |
| Fatty acid-binding protein 5 | FABP5 | 2.203364 | 0.478607 | 0.036643 |
| Aminopeptidase N | ANPEP | 2.199286 | 0.478607 | 0.029536 |
| Cysteine-rich with EGF-like domain protein 1 | CRELD1 | 2.192893 | 0.478607 | 0.044607 |
| Cystatin-M | CST6 | 2.192545 | 0.225225 | 0.039466 |
| Apolipoprotein C-III variant 1 | APOC3 | 2.189622 | 0.151684 | 0.007813 |
| Complement C2 | C2 | 2.186571 | 0.372833 | 0.025504 |
| Adhesion G protein-coupled receptor B3 | ADGRB3 | 2.184578 | 0.241604 | 0.017994 |
| ProSAAS | PCSK1N | 2.176078 | 0.149758 | 0.048488 |
| Protein S100-A6 | S100A6 | 2.173687 | 0.154335 | 0.025605 |
| Serpin B6 | SERPINB6 | 2.170479 | 0.478607 | 0.000202 |
| cDNA FLJ57652, highly similar to Ephrin-A3 | cDNA FLJ57652 | 2.16808 | 0.17876 | 0.008618952 |
| Chloride intracellular channel protein 1 (Chloride channel ABP) | CLIC1 | 2.158926 | 0.478607 | 0.016784 |
| Protein HEG homolog 1 | HEG1 | 2.156519 | 0.161441 | 0.048992 |
| ADP-ribosyl cyclase/cyclic ADP-ribose hydrolase 2 | BST1 | 2.154357 | 0.478607 | 0.007359 |
| Serum albumin | ALB | 2.153855 | 0.478607 | 0.014063 |
| Cocaine- and amphetamine-regulated transcript protein [Cleaved into: CART | CARTPT | 2.147727 | 0.149404 | 0.039617 |
| Seizure 6-like protein 2 | SEZ6L2 | 2.145911 | 0.321825 | 0.002117 |
| Protein TMED7-TICAM2 | TMED7-TICAM2 | 2.142035 | 0.162513 | 0.004032 |
| Sortilin-related receptor | SORL1 | 2.133241 | 0.165384 | 0.043901 |
| Multiple epidermal growth factor-like domains protein 8 | MEGF8 | 2.131211 | 0.166287 | 0.016583 |
| Myocilin | MYOC | 2.101603 | 0.478607 | 0.045514 |
| Putative phospholipase B-like 2 | PLBD2 | 2.098044 | 0.478607 | 0.043347 |
| Lysosomal acid lipase/cholesteryl ester hydrolase | LIPA | 2.045292 | 0.21816 | 0.003679 |
| Angiopoietin-related protein 2 | ANGPTL2 | 2.042996 | 0.478607 | 0.048841 |
| Immunoglobulin heavy variable 3-30-3 | IGHV3-30-3 | 2.042105 | 0.13862 | 0.006048 |
| Ephrin-B1 | EFNB1 | 2.019865 | 0.14488 | 0.036542 |
| Protein delta homolog 2 | DLK2 | 2.000973 | 0.182767 | 0.013962 |
| Metallothionein | MT3 | 2.000279 | 0.145022 | 0.014869 |
