# Supplementary material for: Examination and characterisation of the effect of amitriptyline therapy for chronic neuropathic pain on neuropeptide and proteomic constituents of human cerebrospinal fluid
Source: Brain Behav Immun Health. 2020 Dec 7;10:100184. doi: 10.1016/j.bbih.2020.100184 (PMC8474617; doi:10.1016/j.bbih.2020.100184)
Supplement: Multimedia component 2 [file mmc2.docx]

**Supplement Table 2**: All differentially down-regulated proteins in the responders CSF proteome post treatment (Log fold change (LFC) <-2) in order of log fold change.

| **Proteins** | **Gene** | **LFC** | **LogP** | **FDR** |
| --- | --- | --- | --- | --- |
| Aspartylglucosaminidase | AGA | -12.6602 | 1.771679 | 0.030948 |
| Polypeptide N-acetylgalactosaminyltransferase 2 | GALNT2 | -9.60325 | 1.222043 | 0.037651 |
| Protein shisa-6 | SHISA6 | -8.22558 | 0.975091 | 0.041532 |
| Acyl-CoA-binding protein | DBI | -7.95918 | 0.962663 | 0.003881 |
| Receptor-type tyrosine-protein phosphatase N2 | PTPRN2 | -7.72644 | 0.785049 | 0.044355 |
| Mannan-binding lectin serine protease 1 | MASP1 | -7.32768 | 0.779907 | 0.034526 |
| Peptidyl-prolyl cis-trans isomerase C | PPIC | -7.14398 | 0.9231 | 0.034173 |
| Calmodulin-3 | CALM3 | -7.02432 | 0.852006 | 0.014012 |
| Immunoglobulin kappa variable 3D-11 | IGKV3D-11 | -5.83205 | 0.453133 | 0.003327 |
| Proenkephalin-A | PENK | -5.66517 | 0.417869 | 0.020665 |
| Low affinity immunoglobulin gamma Fc region receptor II-a | FCGR2A | -5.52249 | 0.527208 | 0.028679 |
| Beta-actin-like protein 2 | ACTBL2 | -5.2778 | 0.54097 | 0.040222 |
| IgLON family member 5 | IGLON5 | -5.24902 | 0.49407 | 0.00746 |
| Macrophage colony-stimulating factor 1 | CSF1 | -5.21601 | 0.455243 | 0.027268 |
| Serum amyloid A-1 protein | SAA1 | -5.04779 | 0.820618 | 0.02752 |
| Histone H2B | H2BC15 | -5.03103 | 0.82253 | 0.018145 |
| Spondin-2 | SPON2 | -5.02825 | 0.823643 | 0.010333 |
| Basigin | BSG | -4.971 | 0.823201 | 0.002873 |
| Myelin-associated glycoprotein | MAG | -4.89095 | 0.55983 | 0.030897 |
| Collagen alpha-1(XV) chain | COL15A1 | -4.88832 | 0.409116 | 0.002369 |
| Brain acid soluble protein 1 | BASP1 | -4.85925 | 0.451594 | 0.036341 |
| Glyceraldehyde-3-phosphate dehydrogenase | GAPDH | -4.84919 | 0.682624 | 0.024395 |
| Pyruvate kinase | PKM | -4.83578 | 0.429305 | 0.014919 |
| Immunoglobulin kappa variable 6D-21 | IGKV6D-21 | -4.77294 | 0.42439 | 0.00373 |
| OX-2 membrane glycoprotein | CD200 | -4.74007 | 0.521481 | 0.033821 |
| Multimerin-2 | MMRN2 | -4.73288 | 0.48503 | 0.046673 |
| Renin receptor | ATP6AP2 | -4.72947 | 0.405748 | 0.006754 |
| Mast/stem cell growth factor receptor Kit | KIT | -4.72721 | 0.408195 | 0.028175 |
| Neural cell adhesion molecule 1 | NCAM1 | -4.6395 | 0.392346 | 0.001361 |
| Glutathione S-transferase P | GSTP1 | -4.63039 | 0.407565 | 0.027067 |
| Immunoglobulin heavy variable 3-38 (non-functional) | IGHV3-38 | -4.60007 | 0.473336 | 0.005343 |
| Calcium/calmodulin-dependent protein kinase type II subunit beta | CAMK2B | -4.52398 | 0.392371 | 0.038155 |
| Soluble scavenger receptor cysteine-rich domain-containing protein SSC5D | SSC5D | -4.52263 | 0.539295 | 0.007107 |
| Protein NDRG2 | NDRG2 | -4.50836 | 0.463037 | 0.013458 |
| Nidogen-2 | NID2 | -4.50685 | 0.646147 | 0.038407 |
| Cation-independent mannose-6-phosphate receptor | IGF2R | -4.49586 | 0.548409 | 0.028528 |
| Interleukin-1 receptor accessory protein | IL1RAP | -4.40328 | 0.821889 | 0.047026 |
| Endoplasmin | HSP90B1 | -4.38688 | 0.64174 | 0.029435 |
| Xylosyltransferase 1 | XYLT1 | -4.36094 | 0.823515 | 0.041986 |
| Neurexin-3-beta | NRXN3 | -4.35024 | 0.823702 | 0.004889 |
| Coagulation factor IX | F9 | -4.27474 | 0.453642 | 0.019859 |
| Carbonic anhydrase 4 | CA4 | -4.24828 | 0.349004 | 0.0312 |
| Hemoglobin subunit alpha | HBA1 | -3.47337 | 0.228658 | 0.036139 |
| Glutaminyl-peptide cyclotransferase | QPCT | -3.22511 | 0.571184 | 0.039919 |
| Proliferation marker protein Ki-67 | MKI67 | -3.21954 | 0.217976 | 0.034224 |
| C-reactive protein | CRP | -3.13636 | 0.245939 | 0.022933 |
| Sialate O-acetylesterase | SIAE | -3.09349 | 0.224935 | 0.046724 |
| Thioredoxin | TXN | -3.06449 | 0.244358 | 0.028024 |
| Pyruvate kinase PKM | PKM | -3.03195 | 0.537734 | 0.029335 |
| Immunoglobulin heavy variable 4-4 | IGHV4-4 | -2.96921 | 0.483724 | 0.000857 |
| Double-stranded RNA-specific editase 1 | ADARB1 | -2.94639 | 0.231847 | 0.03624 |
| Immunoglobulin heavy variable 4-4 | IGHV4-4 | -2.85076 | 0.221803 | 0.000857 |
| Fibrillin-1 [Cleaved into: Asprosin] | FBN1 | -2.76768 | 0.204569 | 0.033317 |
| Immunoglobulin superfamily member 8 | IGSF8 | -2.7561 | 0.451409 | 0.009476 |
| Glutamate receptor 4 | GRIA4 | -2.75542 | 0.48125 | 0.013206 |
| Secreted frizzled-related protein 3 | FRZB | -2.74846 | 0.271682 | 0.044052 |
| Gamma-glutamyl hydrolase | GGH | -2.74717 | 0.480914 | 0.044103 |
| Immunoglobulin kappa variable 1-33 | IGKV1-33 | -2.7357 | 0.209784 | 0.020867 |
| Immunoglobulin heavy variable 3-15 | IGHV3-15 | -2.73336 | 0.219064 | 0.004183 |
| Neurexin-1-beta | NRXN1 | -2.70883 | 0.438147 | 0.005645 |
| Aspartate aminotransferase, mitochondrial | GOT2 | -2.6698 | 0.222593 | 0.019556 |
| Cadherin-5 | CDH5 | -2.66691 | 0.262746 | 0.033065 |
| Phosphoinositide-3-kinase-interacting protein 1 | PIK3IP1 | -2.66426 | 0.263597 | 0.044506 |
| Methanethiol oxidase | SELENBP1 | -2.64165 | 0.464427 | 0.037954 |
| Cerebellin-1 | CBLN1 | -2.64047 | 0.465401 | 0.031552 |
| Leptin receptor | LEPR | -2.6207 | 0.478607 | 0.034375 |
| Fc of IgG low affinity IIIa receptor isoform 1 | FCGR3A | -2.61339 | 0.491401 | 0.006956 |
| Aspartate aminotransferase, cytoplasmic | GOT1 | -2.60089 | 0.412672 | 0.030141 |
| MANSC domain-containing protein 1 | MANSC1 | -2.5798 | 0.214056 | 0.046623 |
| Sushi, nidogen and EGF-like domain-containing protein 1 | SNED1 | -2.57015 | 0.26525 | 0.043397 |
| ABHD14A-ACY1 readthrough | ABHD14A-ACY1 | -2.54107 | 0.478607 | 0.006653 |
| Multiple inositol polyphosphate phosphatase 1 | MINPP1 | -2.53832 | 0.193708 | 0.049244 |
| Macrophage mannose receptor 1 | MRC1 | -2.50812 | 0.192127 | 0.031351 |
| Ephrin-A5 | EFNA5 | -2.50339 | 0.478607 | 0.002772 |
| DOMON domain-containing protein FRRS1L | FRRS1L | -2.49743 | 0.18453 | 0.047833 |
| Microtubule-actin cross-linking factor 1, isoforms 1/2/3/5 | MACF1 | -2.49596 | 0.478607 | 0.014819 |
| 45 kDa calcium-binding protein | SDF4 | -2.49579 | 0.246812 | 0.045817 |
| Mesothelin | MSLN | -2.49317 | 0.242061 | 0.014667 |
| Immunoglobulin lambda variable 1-51 | IGLV1-51 | -2.48697 | 0.396368 | 0.021321 |
| Paired immunoglobulin-like type 2 receptor alpha | PILRA | -2.4861 | 0.273984 | 0.009778 |
| NT-3 growth factor receptor | NTRK3 | -2.47886 | 0.222129 | 0.005696 |
| Tyrosine-protein kinase receptor UFO | AXL | -2.47745 | 0.411914 | 0.032863 |
| Hepatocyte growth factor-like protein | MST1 | -2.47145 | 0.26565 | 0.013659 |
| Collagen alpha-3(VI) chain | COL6A3 | -2.46584 | 0.417639 | 0.010938 |
| Peroxiredoxin-2 | PRDX2 | -2.46513 | 0.212381 | 0.033014 |
| Neurogenic locus notch homolog protein 3 | NOTCH3 | -2.44749 | 0.478607 | 0.016683 |
| Dipeptidyl aminopeptidase-like protein 6 | DPP6 | -2.44339 | 0.267497 | 0.011442 |
| Immunoglobulin lambda variable 4-69 | IGLV4-69 | -2.43908 | 0.177116 | 0.000403 |
| Neuromodulin | GAP43 | -2.43437 | 0.478607 | 0.030192 |
| Epsilon-sarcoglycan | SGCE | -2.43282 | 0.410825 | 0.008921 |
| WAP four-disulfide core domain protein 2 | WFDC2 | -2.43186 | 0.218435 | 0.038609 |
| EGF-containing fibulin-like extracellular matrix protein 1 | EFEMP1 | -2.42035 | 0.249294 | 0.00625 |
| HLA class I histocompatibility antigen, Cw-6 alpha chain | HLA-C | -2.41788 | 0.204447 | 0.006452 |
| Receptor-type tyrosine-protein phosphatase-like N | PTPRN | -2.41709 | 0.260107 | 0.03997 |
| Protein Z-dependent protease inhibitor | SERPINA10 | -2.40886 | 0.478607 | 0.013306 |
| Prosaposin receptor GPR37 | GPR37 | -2.39706 | 0.478607 | 0.01754 |
| Alpha-enolase | ENO1 | -2.3931 | 0.23839 | 0.025756 |
| Nucleobindin-2 | NUCB2 | -2.39147 | 0.276203 | 0.00121 |
| Stathmin | STMN1 | -2.38899 | 0.478607 | 0.007157 |
| Tetraspanin | CD81 | -2.38226 | 0.478607 | 0.007611 |
| Hemoglobin subunit delta | HBD | -2.38211 | 0.478607 | 0.022329 |
| Neuroplastin | NPTN | -2.37172 | 0.478607 | 0.048337 |
| Elastin | ELN | -2.36773 | 0.478607 | 0.008367 |
| Ecto-ADP-ribosyltransferase 3 | ART3 | -2.3662 | 0.38671 | 0.038105 |
| Zinc transporter ZIP12 | SLC39A12 | -2.36397 | 0.478607 | 0.040071 |
| Moesin | MSN | -2.36133 | 0.478607 | 0.032006 |
| Fructose-bisphosphate aldolase C | ALDOC | -2.34821 | 0.359878 | 0.027369 |
| Endosialin | CD248 | -2.3466 | 0.478607 | 0.046875 |
| Semaphorin-4B | SEMA4B | -2.33693 | 0.379717 | 0.015625 |
| WASH complex subunit 2A | WASHC2A | -2.33669 | 0.204231 | 0.002016 |
| Carboxypeptidase N catalytic chain | CPN1 | -2.32576 | 0.183881 | 0.029587 |
| Protocadherin-7 | PCDH7 | -2.3168 | 0.478607 | 0.018044 |
| Disintegrin and metalloproteinase domain-containing protein 11 | ADAM11 | -2.31331 | 0.478607 | 0.008417 |
| Carbonic anhydrase 14 | CA14 | -2.31218 | 0.478607 | 0.049042 |
| Prosaposin | PSAP | -2.30855 | 0.478607 | 0.006149 |
| Endonuclease domain-containing 1 protein | ENDOD1 | -2.30357 | 0.357828 | 0.018952 |
| Intercellular adhesion molecule 2 | ICAM2 | -2.29029 | 0.478607 | 0.016028 |
| Reticulon-4 receptor-like 1 | RTN4RL1 | -2.28975 | 0.243539 | 0.041784 |
| Integral membrane protein 2B | ITM2B | -2.28202 | 0.184738 | 0.049597 |
| Cathepsin L1 | CTSL | -2.28035 | 0.378837 | 0.02631 |
| Immunoglobulin heavy variable 3/OR15-7 | IGHV3OR15-7 | -2.27748 | 0.163593 | 0.001109 |
| Alpha-N-acetylgalactosaminidase | NAGA | -2.26812 | 0.478607 | 0.03004 |
| Malectin | MLEC | -2.26753 | 0.478607 | 0.038508 |
| 14-3-3 protein zeta/delta | YWHAZ | -2.26106 | 0.156428 | 0.035938 |
| Lipolysis-stimulated lipoprotein receptor | LSR | -2.24689 | 0.478607 | 0.041935 |
| Complement C1q tumor necrosis factor-related protein 4 | C1QTNF4 | -2.23709 | 0.478607 | 0.046169 |
| Vitamin K-dependent protein Z | PROZ | -2.23593 | 0.254996 | 0.0313 |
| Hepatocyte growth factor activator | HGFAC | -2.20818 | 0.478607 | 0.010282 |
| Transferrin receptor | TFRC | -2.20731 | 0.478607 | 0.013155 |
| UDP-GalNAc:beta-1,3-N-acetylgalactosaminyltransferase 1 | B3GALNT1 | -2.19175 | 0.236203 | 0.018599 |
| 14-3-3 protein gamma | YWHAG | -2.16352 | 0.478607 | 0.035736 |
| Prolargin | PRELP | -2.11238 | 0.341692 | 0.034829 |
| Ephrin-A1 | EFNA1 | -2.10332 | 0.161954 | 0.030847 |
| Carbonic anhydrase 1 | CA1 | -2.03243 | 0.213416 | 0.02006 |
| Cell adhesion molecule 2 | CADM2 | -2.03145 | 0.328332 | 0.042692 |
| Protein FAM19A1 | FAM19A1 | -2.01999 | 0.478607 | 0.041633 |
