# Supplementary material for: Examination and characterisation of the effect of amitriptyline therapy for chronic neuropathic pain on neuropeptide and proteomic constituents of human cerebrospinal fluid
Source: Brain Behav Immun Health. 2020 Dec 7;10:100184. doi: 10.1016/j.bbih.2020.100184 (PMC8474617; doi:10.1016/j.bbih.2020.100184)
Supplement: Multimedia component 3 [file mmc3.docx]

**Supplementary Table 3:** All significantly differentially up-regulated proteins in the non-responders cerebrospinal fluid (CSF) proteome post treatment according to Log fold change (LFC) > 2, in order of LFC

| **Proteins** | **Gene** | **LFC** | **LogP** | **FDR** |
| --- | --- | --- | --- | --- |
| V-type proton ATPase subunit S1 | ATP6AP1 | 17.7330496 | 2.827164629 | 0.000714286 |
| Hemoglobin subunit beta | HBB | 14.7654748 | 0.916853442 | 0.002333333 |
| Phospholipase D4 | PLD4 | 13.82809884 | 1.543076397 | 0.000761905 |
| Polypeptide N-acetylgalactosaminyltransferase 2 | GALNT2 | 13.05085897 | 1.331418633 | 0.001714286 |
| Vitamin K-dependent protein Z | PROZ | 11.81862341 | 1.173749182 | 0.00052381 |
| Coagulation factor XIII B chain | F13B | 11.35208103 | 1.458896657 | 0.00347619 |
| C-X-C motif chemokine 16 | CXCL16 | 10.58946705 | 0.779384114 | 0.005238095 |
| Carbonic anhydrase 1 | CA1 | 10.11483301 | 0.763761581 | 0.001857143 |
| Immunoglobulin lambda variable 6-57 | IGLV6-57 | 9.526642936 | 0.816295313 | 0.001428571 |
| Apolipoprotein B-100 | APOB | 9.459803377 | 0.637670617 | 0.008190476 |
| Transmembrane protein 132D | TMEM132D | 9.338163103 | 0.818080074 | 0.001190476 |
| Growth/differentiation factor 8 | MSTN | 9.13526753 | 0.81789322 | 0.001285714 |
| Calmodulin-3 | CALM3 | 9.092011315 | 0.818125411 | 0.001285714 |
| Phosphoserine aminotransferase | PSAT1 | 8.904575348 | 0.816882924 | 0.001380952 |
| Sodium/potassium-transporting ATPase subunit beta | ATP1B1 | 8.876823153 | 0.819146244 | 0.001095238 |
| Properdin | CFP | 8.741453443 | 0.817537323 | 0.001333333 |
| Immunoglobulin lambda variable 2-14 | IGLV2-14 | 8.534506525 | 0.594337708 | 0.010904762 |
| ProSAAS | PCSK1N | 8.363038199 | 0.573017335 | 0.011761905 |
| Apolipoprotein C-III variant 1 | APOC3 | 8.120662485 | 0.619413957 | 0.009333333 |
| Immunoglobulin heavy variable 3/OR16-12 | IGHV3OR16-12 | 8.118675573 | 0.48248927 | 0.009238095 |
| HCG2044074, isoform CRA_c | MIA-RAB4B | 8.023610932 | 0.613593675 | 0.009761905 |
| Sushi, nidogen and EGF-like domain-containing protein 1 | SNED1 | 7.677152906 | 0.598988898 | 0.010571429 |
| Vitamin D-binding protein | GC | 7.569223949 | 0.548967213 | 0.004095238 |
| Immunoglobulin heavy variable 2-5 | IGHV2-5 | 7.514340264 | 0.468447446 | 0.010619048 |
| Immunoglobulin lambda variable 4-69 | IGLV4-69 | 7.445839678 | 0.443260848 | 0.015809524 |
| Carboxypeptidase N catalytic chain | CPN1 | 7.340732506 | 0.458379973 | 0.01152381 |
| Follistatin-related protein 3 | FSTL3 | 7.336634636 | 0.50099463 | 0.019714286 |
| Ceroid-lipofuscinosis neuronal protein 5 | CLN5 | 7.096825259 | 0.474514729 | 0.010238095 |
| Alpha-actinin-2 | ACTN2 | 7.091871262 | 0.548361172 | 0.004333333 |
| Neuropilin-2 | NRP2 | 7.066779954 | 0.476113873 | 0.01 |
| Protein TMED7-TICAM2 | TMED7-TICAM2 | 7.057989665 | 0.452669959 | 0.011904762 |
| Collagen alpha-1(XV) chain | COL15A1 | 7.056077753 | 0.453962658 | 0.011809524 |
| Glutathione S-transferase P | GSTP1 | 6.920590333 | 0.426606682 | 0.016952381 |
| Angiopoietin-related protein 7 | ANGPTL7 | 6.8380129 | 0.429804911 | 0.016761905 |
| C-reactive protein | CRP | 6.830688136 | 0.419925642 | 0.017428571 |
| Leptin receptor | LEPR | 6.715878623 | 0.548839723 | 0.004190476 |
| Hepatocyte growth factor-like protein | MST1 | 6.650026321 | 0.549628117 | 0.003619048 |
| Ephrin-B1 | EFNB1 | 6.645403181 | 0.411877943 | 0.019333333 |
| Protein CutA (Acetylcholinesterase-associated protein) | CUTA | 6.634366512 | 0.749555549 | 0.016142857 |
| Haptoglobin-related protein | HPR | 6.627066748 | 0.73977755 | 0.016285714 |
| Immunoglobulin kappa variable 1D-16 | IGKV1D-16 | 6.37615994 | 0.548918545 | 0.004142857 |
| Protein disulfide-isomerase A3 | PDIA3 | 6.332456589 | 0.549142891 | 0.003952381 |
| Vitamin K-dependent protein C | PROC | 6.302891254 | 0.811739623 | 0.011095238 |
| Intercellular adhesion molecule 5 | ICAM5 | 6.287291391 | 0.548417995 | 0.004285714 |
| Acid sphingomyelinase-like phosphodiesterase 3b | SMPDL3B | 6.276886259 | 0.549114014 | 0.004 |
| Immunoglobulin heavy variable 3-64 | IGHV3-64 | 6.244223458 | 0.549585862 | 0.003761905 |
| Hemoglobin subunit alpha | HBA1; | 6.229776723 | 0.398118721 | 0.020047619 |
| Apolipoprotein M | APOM | 6.195509093 | 0.730371291 | 0.016619048 |
| Cathepsin F | CTSF | 6.192956924 | 0.549613221 | 0.003666667 |
| Immunoglobulin kappa variable 1-8 | IGKV1-8 | 6.180609635 | 0.830166599 | 0.010190476 |
| Immunoglobulin heavy variable 1-24 | IGHV1-24 | 6.16588865 | 0.549328971 | 0.003809524 |
| Papilin | PAPLN | 6.013988291 | 0.69860971 | 0.01747619 |
| Cartilage oligomeric matrix protein, isoform CRA_b | COMP | 5.987196241 | 0.549152015 | 0.003904762 |
| Endoplasmin | HSP90B1 | 5.907331807 | 0.706684997 | 0.017190476 |
| Beta-1,3-N-acetylglucosaminyltransferase lunatic fringe | LFNG | 5.897992815 | 0.549075965 | 0.004047619 |
| Reticulon-4 receptor | RTN4R | 5.849580765 | 0.682035258 | 0.019238095 |
| Fc of IgG low affinity IIIa receptor isoform 1 | FCGR3A | 5.784267426 | 0.705482828 | 0.017380952 |
| Cerebellin-3 | CBLN3 | 5.761068889 | 0.682742674 | 0.01952381 |
| Alpha-mannosidase 2x | MAN2A2 | 5.740645681 | 0.596976377 | 0.021047619 |
| Immunoglobulin heavy variable 1-69 | IGHV1-69 | 5.641162872 | 0.297208199 | 0.023666667 |
| Angiogenin | ANG | 5.628179073 | 0.63261389 | 0.02047619 |
| Dihydrolipoyl dehydrogenase | DLD | 5.579304831 | 0.549279242 | 0.003857143 |
| Immunoglobulin heavy variable 3-38 (non-functional) | IGHV3-38 | 5.457845075 | 0.312094795 | 0.022952381 |
| Adhesion G protein-coupled receptor L3 | ADGRL3 | 5.283604213 | 0.580763835 | 0.021333333 |
| Ephrin type-B receptor 6 | EPHB6 | 5.271132878 | 0.309321742 | 0.023047619 |
| Peroxiredoxin-6 | PRDX6 | 5.061943463 | 0.293290095 | 0.022238095 |
| Immunoglobulin kappa variable 2-24 | IGKV2-24 | 5.026254041 | 0.270859514 | 0.024904762 |
| Proenkephalin-A | PENK | 4.780188833 | 0.233363727 | 0.02747619 |
| Neuron-specific vesicular protein calcyon | CALY | 4.750963688 | 0.272408083 | 0.023 |
| Antileukoproteinase | SLPI | 4.5691826 | 0.256134763 | 0.025761905 |
| Collagen alpha-2 | COL4A2 | 4.545761926 | 0.264332197 | 0.02547619 |
| Hemoglobin subunit delta | HBD | 4.37774631 | 0.319667337 | 0.013761905 |
| Cystatin-M | CST6 | 4.310260705 | 0.246172019 | 0.026619048 |
| Collagen alpha-2 | COL6A2 | 4.304086753 | 0.254459302 | 0.025904762 |
| Sialic acid-binding Ig-like lectin 14 | SIGLEC14 | 4.262669018 | 0.25365606 | 0.024142857 |
| Carbonic anhydrase 2 | CA2 | 4.197131838 | 0.319667337 | 0.013857143 |
| Tyrosine-protein kinase receptor TYRO3 | TYRO3 | 4.188203812 | 0.223234079 | 0.026285714 |
| Alpha-enolase | ENO1 | 4.182824748 | 0.227350017 | 0.027761905 |
| Fibroblast growth factor receptor 3 | FGFR3 | 4.164783137 | 0.244335446 | 0.026761905 |
| Calcium/calmodulin-dependent protein kinase type II subunit beta | CAMK2B | 4.128267901 | 0.238390228 | 0.027190476 |
| Catalase | CAT | 4.126898902 | 0.319667337 | 0.013666667 |
| Sodium channel subunit beta-3 | SCN3B | 4.075798852 | 0.261737055 | 0.023714286 |
| Cell growth regulator with EF hand domain protein 1 | CGREF1 | 4.070104463 | 0.2191075 | 0.026857143 |
| Flavin reductase | BLVRB | 4.069440024 | 0.319667337 | 0.013 |
| NT-3 growth factor receptor | NTRK3 | 4.067756925 | 0.253342166 | 0.024238095 |
| Hemoglobin subunit gamma-2 | HBG2 | 3.915710722 | 0.319667337 | 0.012857143 |
| Proliferation marker protein Ki-67 | MKI67 | 3.892976216 | 0.319667337 | 0.012952381 |
| Neuroligin-2 | NLGN2 | 3.880229269 | 0.219425119 | 0.026714286 |
| Immunoglobulin lambda variable 2-18 | IGLV2-18 | 3.872142928 | 0.218576646 | 0.026952381 |
| Carbonic anhydrase 3 | CA3 | 3.850760324 | 0.319667337 | 0.01347619 |
| Calcium/calmodulin-dependent protein kinase type II subunit alpha | CAMK2A | 3.804756505 | 0.211397801 | 0.027428571 |
| Glutathione hydrolase 7 | GGT7 | 3.798224994 | 0.225899494 | 0.026142857 |
| Alpha-1-antitrypsin | SERPINA1 | 3.754103048 | 0.189456477 | 0.02847619 |
| Ankyrin-1 | ANK1 | 3.736327853 | 0.319667337 | 0.013190476 |
| Immunoglobulin lambda variable 3-9 | IGLV3-9 | 3.682708127 | 0.199172748 | 0.027952381 |
| Phosphoglycerate mutase 1 | PGAM1 | 3.677274295 | 0.215181944 | 0.027 |
| Nucleoside diphosphate kinase A | NME1 | 3.6193864 | 0.319667337 | 0.013238095 |
| Bisphosphoglycerate mutase | BPGM | 3.610139029 | 0.319667337 | 0.013428571 |
| Adenylate kinase isoenzyme 1 | AK1 | 3.56142698 | 0.319667337 | 0.013904762 |
| Titin | TTN | 3.507405622 | 0.187706448 | 0.028666667 |
| Delta-aminolevulinic acid dehydratase | ALAD | 3.498456138 | 0.319667337 | 0.013285714 |
| Eukaryotic translation initiation factor 5A | EIF5A2 | 3.474555969 | 0.319667337 | 0.014714286 |
| Purine nucleoside phosphorylase (PNP) | PNP | 3.43343108 | 0.319667337 | 0.013952381 |
| Creatine kinase M-type | CKM | 3.422876903 | 0.319667337 | 0.01352381 |
| Hsc70-interacting protein | ST13 | 3.410878045 | 0.319667337 | 0.014380952 |
| Spectrin beta chain, erythrocytic | SPTB | 3.389299938 | 0.319667337 | 0.013380952 |
| Immunoglobulin heavy variable 4-34 | IGHV4-34 | 3.377413886 | 0.207841224 | 0.02852381 |
| Msx2-interacting protein | SPEN | 3.37459319 | 0.319667337 | 0.01247619 |
| Erythrocyte membrane protein band 4.2 | EPB42 | 3.335313252 | 0.319667337 | 0.013142857 |
| Retinal dehydrogenase 1 | ALDH1A1 | 3.313064575 | 0.319667337 | 0.014 |
| Band 3 anion transport protein | SLC4A1 | 3.306749616 | 0.319667337 | 0.013714286 |
| Cerebellin-2 | CBLN2 | 3.284041132 | 0.184522015 | 0.028809524 |
| Immunoglobulin kappa variable 1-12 | IGKV1-12 | 3.274879456 | 0.319667337 | 0.015190476 |
| Myosin-7 | MYH7 | 3.256953376 | 0.319667337 | 0.013333333 |
| Moesin | MSN | 3.242891312 | 0.319667337 | 0.013047619 |
| Hemoglobin subunit zeta | HBZ | 3.226990564 | 0.319667337 | 0.013809524 |
| Sema domain, transmembrane domain (TM), and cytoplasmic domain, (Semaphorin) 6A, isoform CRA_d (Semaphorin-6A) | SEMA6A | 3.215990067 | 0.319667337 | 0.015380952 |
| Reticulon-4 receptor-like 1 | RTN4RL1 | 3.2011043 | 0.319667337 | 0.012619048 |
| Anthrax toxin receptor 1 | ANTXR1 | 3.186499187 | 0.319667337 | 0.012285714 |
| Prosaposin | PSAP | 3.186161041 | 0.319667337 | 0.015095238 |
| GTP-binding nuclear protein Ran | RAN | 3.180057798 | 0.319667337 | 0.014761905 |
| Nesprin-2 | SYNE2 | 3.160519464 | 0.319667337 | 0.015333333 |
| Protein 4.1 | EPB41 | 3.151472364 | 0.319667337 | 0.015047619 |
| Chitinase domain-containing protein 1 | CHID1 | 3.134127208 | 0.319667337 | 0.012380952 |
| Vimentin variant 3 | VIM | 3.13274874 | 0.319667337 | 0.014952381 |
| Biglycan | BGN | 3.127339499 | 0.319667337 | 0.013095238 |
| Voltage-dependent calcium channel subunit alpha-2/delta-2 | CACNA2D2 | 3.119617735 | 0.319667337 | 0.014666667 |
| Soluble scavenger receptor cysteine-rich domain-containing protein SSC5D | SSC5D | 3.114161287 | 0.187681593 | 0.028619048 |
| Intercellular adhesion molecule 2 | ICAM2 | 3.098848343 | 0.319667337 | 0.014142857 |
| Mesothelin | MSLN | 3.098525728 | 0.319667337 | 0.014285714 |
| Alpha-N-acetylglucosaminidase | NAGLU | 3.08029529 | 0.319667337 | 0.012904762 |
| Protein S100-A9 | S100A9 | 3.07059506 | 0.319667337 | 0.013571429 |
| Lactotransferrin | LTF | 3.060737882 | 0.319667337 | 0.014571429 |
| Rho GTPase-activating protein 5 | ARHGAP5 | 3.059758323 | 0.319667337 | 0.015285714 |
| Semaphorin-4D | SEMA4D | 3.052460534 | 0.319667337 | 0.01452381 |
| Elastin | ELN | 3.050208228 | 0.319667337 | 0.014809524 |
| Folate receptor beta | FOLR2 | 3.039188521 | 0.194904771 | 0.029190476 |
| Chondroitin sulfate proteoglycan 4 | CSPG4 | 3.033057077 | 0.319667337 | 0.012666667 |
| Matrix remodeling-associated protein 8 | MXRA8 | 3.028824125 | 0.319667337 | 0.012428571 |
| Immunoglobulin lambda variable 3-25 | IGLV3-25 | 3.023512363 | 0.181672588 | 0.029571429 |
| Osteoclast-associated immunoglobulin-like receptor | OSCAR | 3.014577048 | 0.319667337 | 0.015571429 |
| Nebulin | NEB | 3.009337561 | 0.319667337 | 0.015428571 |
| HCG2044781 (TMEM189-UBE2V1 readthrough) | TMEM189-UBE2V1 | 2.960947037 | 0.319667337 | 0.014190476 |
| Apolipoprotein F | APOF | 2.953632627 | 0.319667337 | 0.012761905 |
| Integral membrane protein DGCR2/IDD | DGCR2 | 2.950370244 | 0.319667337 | 0.014428571 |
| Palmitoyl-protein thioesterase 1 | PPT1 | 2.942332132 | 0.319667337 | 0.01447619 |
| Ribose-phosphate pyrophosphokinase 1 | PRPS1 | 2.942299434 | 0.319667337 | 0.014857143 |
| Target of Nesh-SH3 | ABI3BP | 2.938692365 | 0.319667337 | 0.014619048 |
| Complement C2 | C2 | 2.933782373 | 0.186988089 | 0.029380952 |
| Cholinesterase | BCHE | 2.929139001 | 0.319667337 | 0.013619048 |
| Interleukin-1 receptor accessory protein | IL1RAP | 2.916219984 | 0.319667337 | 0.012238095 |
| Zona pellucida sperm-binding protein 2 | ZP2 | 2.903082166 | 0.319667337 | 0.012809524 |
| Mast/stem cell growth factor receptor Kit | KIT | 2.902766228 | 0.195698071 | 0.029095238 |
| Immunoglobulin heavy variable 3-9 | IGHV3-9 | 2.886199747 | 0.163831344 | 0.030809524 |
| Leukocyte-associated immunoglobulin-like receptor 1 | LAIR1 | 2.847871235 | 0.319667337 | 0.015142857 |
| Membrane-associated progesterone receptor component 1 (mPR) | PGRMC1 | 2.8319664 | 0.319667337 | 0.014095238 |
| Dyslexia-associated protein KIAA0319 | KIAA0319 | 2.827532904 | 0.319667337 | 0.012714286 |
| Procollagen-lysine,2-oxoglutarate 5-dioxygenase 3 | PLOD3 | 2.812179838 | 0.319667337 | 0.014238095 |
| EPHB2 protein (Ephrin type-B receptor 2) | EPHB2 | 2.810941424 | 0.319667337 | 0.014904762 |
| Complement C1q tumor necrosis factor-related protein 4 | C1QTNF4 | 2.800175803 | 0.319667337 | 0.012333333 |
| Cysteine-rich with EGF-like domain protein 1 | CRELD1 | 2.788597379 | 0.319667337 | 0.01252381 |
| ADAM DEC1 | ADAMDEC1 | 2.785990579 | 0.319667337 | 0.014047619 |
| Immunoglobulin lambda variable 3-19 | IGLV3-19 | 2.683903899 | 0.156561474 | 0.031809524 |
| Angiopoietin-related protein 2 | ANGPTL2 | 2.639735631 | 0.319667337 | 0.012190476 |
| Immunoglobulin heavy variable 1/OR15-1 | IGHV1OR15-1 | 2.627850056 | 0.111442676 | 0.034190476 |
| ADP-ribosyl cyclase/cyclic ADP-ribose hydrolase 2 | BST1 | 2.603515625 | 0.319667337 | 0.015 |
| Peptidyl-prolyl cis-trans isomerase A | PPIA | 2.589903014 | 0.16918915 | 0.030142857 |
| Ephrin-B2 | EFNB2 | 2.520082542 | 0.157990932 | 0.031666667 |
| Neuroendocrine protein 7B2 | SCG5 | 2.501035418 | 0.15461686 | 0.032 |
| Peptidyl-prolyl cis-trans isomerase B | PPIB | 2.491742202 | 0.166194609 | 0.030333333 |
| Immunoglobulin heavy variable 3-64D | IGHV3-64D | 2.470560755 | 0.147379086 | 0.032619048 |
| Protocadherin-1 | PCDH1 | 2.434499877 | 0.162878092 | 0.031047619 |
| Protein S100-A6 | S100A6 | 2.319936889 | 0.106941093 | 0.034619048 |
| Matrix Gla protein | MGP | 2.26031174 | 0.127735938 | 0.034047619 |
| N | AGA | 2.244946889 | 0.143252089 | 0.032761905 |
| Cochlin | COCH | 2.198242051 | 0.133661232 | 0.033571429 |
| Podocalyxin-like protein 2 | PODXL2 | 2.176144532 | 0.138252377 | 0.033238095 |
| Zinc transporter ZIP10 | SLC39A10 | 2.143930095 | 0.134474966 | 0.033428571 |
| Vascular cell adhesion protein 1 | VCAM1 | 2.122811113 | 0.133555711 | 0.033619048 |
| Insulin-like growth factor binding protein 3 isoform b | IGFBP3 | 2.117982251 | 0.102720954 | 0.034904762 |
| Laminin subunit gamma-1 | LAMC1 | 2.06313324 | 0.133007719 | 0.033761905 |
| Sialate O-acetylesterase | SIAE | 2.054455621 | 0.127116763 | 0.034142857 |
| UPF0606 protein KIAA1549L | KIAA1549L | 2.037216255 | 0.115539061 | 0.034809524 |
| Immunoglobulin heavy variable 1-46 | IGHV1-46 | 2.035560949 | 0.10115746 | 0.035 |
| Immunoglobulin lambda constant 3 | IGLC3 | 2.032724108 | 0.092295332 | 0.035380952 |
