# Supplementary material for: Examination and characterisation of the effect of amitriptyline therapy for chronic neuropathic pain on neuropeptide and proteomic constituents of human cerebrospinal fluid
Source: Brain Behav Immun Health. 2020 Dec 7;10:100184. doi: 10.1016/j.bbih.2020.100184 (PMC8474617; doi:10.1016/j.bbih.2020.100184)
Supplement: Multimedia component 4 [file mmc4.docx]

**Supplementary Table 4:** All significantly differentially down-regulated proteins in the non-responders cerebrospinal fluid (CSF) proteome post treatment according to Log fold change (LFC) < -2, in order of LFC

| **Proteins** | **Gene** | **LFC** | **LogP** | **FDR** |
| --- | --- | --- | --- | --- |
| Double-stranded RNA-specific editase 1 | ADARB1 | -17.2087869 | 1.727415111 | 9.52381E-05 |
| Heat shock cognate 71 kDa protein | HSPA8 | -15.82209035 | 1.612765452 | 0.000142857 |
| Mannan-binding lectin serine protease 1 | MASP1 | -15.26076494 | 1.74757113 | 4.7619E-05 |
| Protein FAM19A5 | TAFA5 | -14.02971186 | 1.201375765 | 0.000428571 |
| Serotransferrin | TF | -13.95079844 | 1.289981347 | 0.000285714 |
| Contactin-4 | CNTN4 | -12.88501344 | 1.219143136 | 0.000380952 |
| Netrin-G1 | NTNG1 | -12.74417686 | 1.130274283 | 0.000571429 |
| Immunoglobulin kappa variable 1-33 | IGKV1-33 | -12.69562006 | 1.036308659 | 0.000666667 |
| Contactin-6 | CNTN6 | -12.64343766 | 1.179428872 | 0.00047619 |
| Semaphorin-3G | SEMA3G | -12.44467851 | 1.288608643 | 0.001809524 |
| V-set and immunoglobulin domain-containing protein 4 | VSIG4 | -12.37137794 | 1.112227695 | 0.000619048 |
| Beta-actin-like protein 2 | ACTBL2 | -11.97152758 | 1.394164245 | 0.004809524 |
| Immunoglobulin kappa variable 3D-11 | IGKV3D-11 | -11.83203895 | 0.79544591 | 0.004666667 |
| Ephrin type-A receptor 5 | EPHA5 | -11.02044582 | 1.393979428 | 0.004904762 |
| alpha-1,2-Mannosidase | MAN1B1 | -10.86874819 | 1.394104693 | 0.004857143 |
| Xyloside xylosyltransferase 1 | XXYLT1 | -10.64442716 | 0.862217455 | 0.003238095 |
| Myelin-associated glycoprotein | MAG | -10.55789995 | 1.393460684 | 0.004952381 |
| ZNF511-PRAP1 readthrough | ZNF511-PRAP1 | -10.49814292 | 0.789756711 | 0.005095238 |
| C-type mannose receptor 2 | MRC2 | -10.42917606 | 0.786195248 | 0.005047619 |
| Netrin receptor DCC | DCC | -10.36592538 | 0.807453693 | 0.00452381 |
| SLIT and NTRK-like protein 5 | SLITRK5 | -10.33363192 | 0.738262511 | 0.005761905 |
| Fibrillin-1 [Cleaved into: Asprosin] | FBN1 | -10.29858828 | 0.751509035 | 0.005666667 |
| Transmembrane glycoprotein NMB | GPNMB | -10.25154924 | 1.390688251 | 0.005142857 |
| Spectrin alpha chain, erythrocytic 1 | SPTA1 | -10.132967 | 1.390104493 | 0.005190476 |
| Glypican-1 [Cleaved into: Secreted glypican-1] | GPC1 | -9.970864228 | 0.802395174 | 0.00152381 |
| Peptidyl-prolyl cis-trans isomerase C | PPIC | -9.96184894 | 0.775571258 | 0.005380952 |
| Adhesion G protein-coupled receptor B1 | ADGRB1 | -9.916713238 | 1.394168899 | 0.004761905 |
| Serine protease inhibitor Kazal-type 6 | SPINK6 | -9.858383724 | 0.793315005 | 0.001619048 |
| Secreted frizzled-related protein 4 | SFRP4 | -9.847377573 | 0.883573297 | 0.000857143 |
| Protein HEG homolog 1 | HEG1 | -9.568422726 | 0.733865457 | 0.005857143 |
| Cholecystokinin | CCK | -9.546958923 | 0.734031886 | 0.002190476 |
| Beta-mannosidase | MANBA | -9.504348891 | 0.829070518 | 0.001047619 |
| Mitotic spindle assembly checkpoint protein MAD1 | MAD1L1 | -9.424930232 | 0.670643783 | 0.008571429 |
| Protocadherin-9 | PCDH9 | -9.320859909 | 0.794408112 | 0.001571429 |
| Contactin-associated protein-like 2 | CNTNAP2 | -9.249386719 | 0.708944299 | 0.002285714 |
| Growth hormone A1 | PRL | -8.442205565 | 0.670371435 | 0.008619048 |
| Testican-3 | SPOCK3 | -8.427104201 | 0.714164085 | 0.002238095 |
| DOMON domain-containing protein FRRS1L | FRRS1L | -8.412509441 | 0.640966018 | 0.008952381 |
| Polypeptide N-acetylgalactosaminyltransferase 6 | GALNT6 | -8.328299318 | 0.555926954 | 0.008095238 |
| WAP four-disulfide core domain protein 1 | WFDC1 | -8.223711831 | 0.623258688 | 0.009571429 |
| Protein delta homolog 2 | DLK2 | -7.966972283 | 0.739793262 | 0.002142857 |
| VPS10 domain-containing receptor SorCS1 | SORCS1 | -7.924479553 | 0.626653432 | 0.009285714 |
| Protocadherin-17 | PCDH17 | -7.780732155 | 0.661996425 | 0.008761905 |
| Extracellular serine/threonine protein kinase FAM20C | FAM20C | -7.74975041 | 0.612149255 | 0.009809524 |
| Cadherin-18 | CDH18 | -7.740121569 | 0.652409163 | 0.00252381 |
| Plexin domain-containing protein 1 | PLXDC1 | -7.733608314 | 0.611191396 | 0.009904762 |
| Protein FAM69C | DIPK1C | -7.612144675 | 0.478729287 | 0.010666667 |
| Complement C1q tumor necrosis factor-related protein 5 | C1QTNF5 | -7.607075146 | 0.599873179 | 0.010380952 |
| Protein ERGIC-53 | LMAN1 | -7.604011944 | 0.464996062 | 0.011333333 |
| Dyslexia-associated protein KIAA0319-like protein | KIAA0319L | -7.50304372 | 0.582919765 | 0.010714286 |
| Beta-hexosaminidase subunit beta | HEXB | -7.495227337 | 0.631250613 | 0.002952381 |
| Lysosomal alpha-glucosidase | GAA | -7.476088864 | 0.632080457 | 0.009190476 |
| A disintegrin and metalloproteinase with thrombospondin motifs 4 | ADAMTS4 | -7.46701295 | 0.564688679 | 0.011190476 |
| Sia-alpha-2,3-Gal-beta-1,4-GlcNAc-R:alpha 2,8-sialyltransferase | ST8SIA3 | -7.439700603 | 0.500696171 | 0.009619048 |
| Tropomyosin alpha-4 chain | TPM4 | -7.338697093 | 0.422970285 | 0.01652381 |
| Junctional adhesion molecule B | JAM2 | -7.230527333 | 0.607152028 | 0.010095238 |
| Low-density lipoprotein receptor-related protein 11 | LRP11 | -7.207210677 | 0.608560226 | 0.009952381 |
| Netrin receptor UNC5C | UNC5C | -7.205382483 | 0.592922124 | 0.01047619 |
| Cadherin-11 | CDH11 | -7.190963405 | 0.573548576 | 0.010952381 |
| Calnexin | CANX | -7.155601433 | 0.603356187 | 0.010142857 |
| Tetratricopeptide repeat domain 7A, isoform CRA_a | TTC7A | -7.111987659 | 0.452827415 | 0.011952381 |
| NAD | NAXE | -7.070923397 | 0.616859375 | 0.009714286 |
| Neuropilin-1 | NRP1 | -7.046418122 | 0.488470551 | 0.010333333 |
| Follistatin-related protein 5 | FSTL5 | -7.005941868 | 0.417417806 | 0.016857143 |
| Glia-derived nexin | SERPINE2 | -6.969583035 | 0.424984083 | 0.016380952 |
| Calsyntenin-3 | CLSTN3 | -6.921108382 | 0.606538174 | 0.003 |
| Metallothionein | MT3 | -6.91421536 | 0.435302622 | 0.016 |
| Ig-like domain-containing protein | n/a | -6.868158 | 0.530219205 | 0.005285714 |
| Scrapie-responsive protein 1 | SCRG1 | -6.863022259 | 0.440962373 | 0.015761905 |
| Xylosyltransferase 1 | XYLT1 | -6.831765652 | 0.588860966 | 0.01052381 |
| Chordin-like protein 1 | CHRDL1 | -6.777474744 | 0.559736056 | 0.003333333 |
| Receptor-type tyrosine-protein phosphatase N2 | PTPRN2 | -6.776586192 | 0.560417428 | 0.003380952 |
| Aspartate aminotransferase, mitochondrial | GOT2 | -6.7267719 | 0.42946567 | 0.016238095 |
| Acid ceramidase | ASAH1 | -6.725760392 | 0.593715894 | 0.003142857 |
| Protein shisa-6 | SHISA6 | -6.723671777 | 0.392282934 | 0.019428571 |
| Low affinity immunoglobulin gamma Fc region receptor II-a | FCGR2A | -6.670365402 | 0.559488062 | 0.003428571 |
| Guanine deaminase | GDA | -6.627694471 | 0.563704334 | 0.003285714 |
| Chordin | CHRD | -6.598666668 | 0.594833754 | 0.003095238 |
| Polypeptide N-acetylgalactosaminyltransferase | GALNT7 | -6.543560982 | 0.409474884 | 0.017095238 |
| Out at first protein homolog | OAF | -6.524238041 | 0.514026478 | 0.005714286 |
| Beta-1,4-galactosyltransferase 1 | B4GALT1 | -6.496834959 | 0.394128656 | 0.019285714 |
| Golgi integral membrane protein 4 | GOLIM4 | -6.399989196 | 0.530323566 | 0.005333333 |
| Serum albumin | ALB | -6.398368563 | 0.544868944 | 0.004428571 |
| Delta and Notch-like epidermal growth factor-related receptor | DNER | -6.356620584 | 0.496585467 | 0.006047619 |
| Ryanodine receptor 2 | RYR2 | -6.338811874 | 0.698114856 | 0.018666667 |
| Cadherin-6 | CDH6 | -6.336361613 | 0.549470216 | 0.004238095 |
| Phospholipase D3 | PLD3 | -6.282430989 | 0.503528779 | 0.005952381 |
| Hypoxia up-regulated protein 1 | HYOU1 | -6.200163228 | 0.490842118 | 0.006285714 |
| Immunoglobulin heavy variable 3-15 | IGHV3-15 | -6.137991973 | 0.499365413 | 0.006 |
| Protein CASC4 | GOLM2 | -6.110157967 | 0.698114856 | 0.018761905 |
| Endoplasmic reticulum aminopeptidase 1 | ERAP1 | -6.003855228 | 0.698114856 | 0.017809524 |
| Growth arrest-specific protein 6 | GAS6 | -5.942723751 | 0.489024445 | 0.006380952 |
| Cadherin-5 | CDH5 | -5.936657906 | 0.698114856 | 0.018190476 |
| Thioredoxin | TXN | -5.915117264 | 0.49022205 | 0.006333333 |
| Selenoprotein M | SELENOM | -5.728058338 | 0.698114856 | 0.019095238 |
| Macrophage mannose receptor 1 | MRC1 | -5.691224711 | 0.472828649 | 0.006619048 |
| Neural cell adhesion molecule 1 | NCAM1 | -5.689100197 | 0.445121602 | 0.006952381 |
| Receptor-type tyrosine-protein phosphatase-like N | PTPRN | -5.686585903 | 0.698114856 | 0.018 |
| Coactosin-like protein | COTL1 | -5.538314819 | 0.698114856 | 0.018095238 |
| Neural cell adhesion molecule L1-like protein | CHL1 | -5.511045865 | 0.47250192 | 0.006666667 |
| Forkhead-associated domain-containing protein 1 | FHAD1 | -5.469340801 | 0.698114856 | 0.018952381 |
| Lysosomal acid lipase/cholesteryl ester hydrolase | LIPA | -5.443239689 | 0.698114856 | 0.019047619 |
| Plastin-2 | LCP1 | -5.415706294 | 0.447918598 | 0.006857143 |
| Malectin | MLEC | -5.380572796 | 0.698114856 | 0.018047619 |
| Cation-independent mannose-6-phosphate receptor | IGF2R | -5.369415283 | 0.698114856 | 0.018380952 |
| Butyrophilin subfamily 2 member A1 | BTN2A1 | -5.350942612 | 0.698114856 | 0.018857143 |
| Immunoglobulin lambda variable 4-60 | IGLV4-60 | -5.324790955 | 0.698114856 | 0.019142857 |
| Neuromodulin | GAP43 | -5.279493332 | 0.698114856 | 0.018333333 |
| Microtubule-actin cross-linking factor 1, isoforms 1/2/3/5 | MACF1 | -5.275220871 | 0.698114856 | 0.018714286 |
| Neuroplastin | NPTN | -5.255766392 | 0.698114856 | 0.017714286 |
| UPF0454 protein C12orf49 | C12orf49 | -5.254859447 | 0.698114856 | 0.017857143 |
| C-type natriuretic peptide [Cleaved into: CNP-22;CNP-29;CNP-53] | NPPC | -5.234627656 | 0.293988004 | 0.024047619 |
| Calsyntenin-2 | CLSTN2 | -5.21847868 | 0.698114856 | 0.017904762 |
| Sodium/potassium-transporting ATPase subunit alpha | ATP1A2 | -5.195561886 | 0.698114856 | 0.018904762 |
| UDP-GalNAc:beta-1,3-N-acetylgalactosaminyltransferase 1 | B3GALNT1 | -5.175493717 | 0.698114856 | 0.01847619 |
| Macrophage colony-stimulating factor 1 | CSF1 | -5.156295844 | 0.289769206 | 0.023095238 |
| Contactin-3 | CNTN3 | -5.13400507 | 0.698114856 | 0.017761905 |
| Immunoglobulin lambda variable 1-44 | IGLV1-44 | -5.103190899 | 0.698114856 | 0.018428571 |
| Immunoglobulin heavy variable 3-20 | IGHV3-20 | -5.087020874 | 0.698114856 | 0.019 |
| Transmembrane protein 59-like | TMEM59L | -5.066848755 | 0.698114856 | 0.017666667 |
| Semaphorin-6D | SEMA6D | -5.049332346 | 0.310819284 | 0.023428571 |
| Sodium/iodide cotransporter | SLC5A5 | -5.01409483 | 0.698114856 | 0.017952381 |
| Chondroadherin | CHAD | -5.004061222 | 0.698114856 | 0.01852381 |
| Non-secretory ribonuclease | RNASE2 | -4.991362504 | 0.285910962 | 0.024428571 |
| Sex hormone-binding globulin, isoform CRA_a | SHBG | -4.991299357 | 0.301862823 | 0.023809524 |
| Connective tissue growth factor | CCN2 | -4.912896156 | 0.698114856 | 0.018238095 |
| Alpha-1,3-mannosyl-glycoprotein 2-beta-N-acetylglucosaminyltransferase | MGAT1 | -4.838296618 | 0.284405125 | 0.02447619 |
| WW domain-binding protein 2 | WBP2 | -4.815889495 | 0.274800999 | 0.024761905 |
| Chloride intracellular channel protein 1 (Chloride channel ABP) | CLIC1 | -4.774667263 | 0.698114856 | 0.018571429 |
| Ephrin-A1 | EFNA1 | -4.738621099 | 0.285836522 | 0.024380952 |
| Procollagen-lysine,2-oxoglutarate 5-dioxygenase 1 | PLOD1 | -4.710621357 | 0.698114856 | 0.018142857 |
| Immunoglobulin kappa variable 2-29 | IGKV2-29 | -4.700024128 | 0.238457201 | 0.02552381 |
| Plasma alpha-L-fucosidase | FUCA2 | -4.669029372 | 0.251796477 | 0.025666667 |
| Aminopeptidase | NPEPPS | -4.644252777 | 0.698114856 | 0.018809524 |
| Protocadherin gamma-C5 | PCDHGC5 | -4.639968804 | 0.260880522 | 0.025333333 |
| Cleavage stimulation factor subunit 3 | CSTF3 | -4.589632239 | 0.432178734 | 0.007142857 |
| Beta-Ala-His dipeptidase | CNDP1 | -4.500663417 | 0.22492373 | 0.005619048 |
| Leucine-rich repeat transmembrane neuronal protein 2 | LRRTM2 | -4.458975315 | 0.278282867 | 0.024619048 |
| Multiple inositol polyphosphate phosphatase 1 | MINPP1 | -4.441134589 | 0.442359689 | 0.007047619 |
| WASH complex subunit 2A | WASHC2A | -4.435419559 | 0.263168602 | 0.025142857 |
| Testican-2 | SPOCK2 | -4.377730506 | 0.230407634 | 0.025857143 |
| Adhesion G protein-coupled receptor B2 | ADGRB2 | -4.325736795 | 0.242171746 | 0.025238095 |
| WAP four-disulfide core domain protein 2 | WFDC2 | -4.302537509 | 0.263999194 | 0.025047619 |
| Immunoglobulin kappa variable 6D-21 | IGKV6D-21 | -4.280338969 | 0.245267851 | 0.026 |
| Transmembrane protein 132A | TMEM132A | -4.280067171 | 0.418484252 | 0.007190476 |
| Sulfhydryl oxidase 2 | QSOX2 | -4.225917203 | 0.240475501 | 0.025380952 |
| Immunoglobulin lambda-like polypeptide 1 | IGLL1 | -4.2237057 | 0.255245854 | 0.025571429 |
| Coagulation factor IX | F9 | -4.21966832 | 0.241075193 | 0.025285714 |
| Thymosin beta-4 | TMSB4X | -4.113992419 | 0.213201816 | 0.027095238 |
| Neural cell adhesion molecule L1 | L1CAM | -4.100632668 | 0.389830311 | 0.007952381 |
| Adhesion G protein-coupled receptor L1 | ADGRL1 | -4.093407699 | 0.390182922 | 0.007857143 |
| Rab GDP dissociation inhibitor alpha | GDI1 | -4.079544067 | 0.229215702 | 0.025952381 |
| Neurexophilin-1 | NXPH1 | -3.999327932 | 0.206137593 | 0.02752381 |
| Basal cell adhesion molecule | BCAM | -3.991728783 | 0.253381843 | 0.025619048 |
| Adipocyte enhancer-binding protein 1 | AEBP1 | -3.945445946 | 0.372211016 | 0.008380952 |
| Thrombospondin-4 | THBS4 | -3.940882887 | 0.221122464 | 0.02652381 |
| Prolow-density lipoprotein receptor-related protein 1 | LRP1 | -3.923822335 | 0.377220948 | 0.008285714 |
| Polypeptide N-acetylgalactosaminyltransferase 18 | GALNT18 | -3.848302841 | 0.236403787 | 0.026571429 |
| Protocadherin Fat 2 | FAT2 | -3.741374016 | 0.329352672 | 0.011714286 |
| Glyceraldehyde-3-phosphate dehydrogenase | GAPDH | -3.723533358 | 0.329116956 | 0.011571429 |
| Immunoglobulin superfamily member 21 | IGSF21 | -3.702085291 | 0.209832952 | 0.027285714 |
| Follistatin-related protein 4 | FSTL4 | -3.68143865 | 0.338088026 | 0.010809524 |
| Heparan-sulfate 6-O-sulfotransferase 3 | HS6ST3 | -3.672327246 | 0.370803359 | 0.00847619 |
| Neuronal pentraxin-2 | NPTX2 | -3.672268867 | 0.345037673 | 0.010285714 |
| Inter-alpha-trypsin inhibitor heavy chain H5 | ITIH5 | -3.636583737 | 0.318235533 | 0.015714286 |
| L-selectin | SELL | -3.589487825 | 0.347736489 | 0.009857143 |
| Golgi membrane protein 1 | GOLM1 | -3.57973378 | 0.339495427 | 0.010857143 |
| Acyl-CoA-binding protein | DBI | -3.559237821 | 0.35649969 | 0.009047619 |
| Neural proliferation differentiation and control protein 1 | NPDC1 | -3.54023041 | 0.337655672 | 0.010761905 |
| Roundabout homolog 1 | ROBO1 | -3.53718601 | 0.330444244 | 0.011380952 |
| Complement C1q subcomponent subunit A | C1QA | -3.494327273 | 0.306991639 | 0.016333333 |
| Neutral alpha-glucosidase AB | GANAB | -3.450649534 | 0.356923238 | 0.009142857 |
| SPARC-related modular calcium-binding protein 1 | SMOC1 | -3.439652034 | 0.335902281 | 0.011 |
| Mannosyl-oligosaccharide 1,2-alpha-mannosidase IA | MAN1A1 | -3.43908017 | 0.312073977 | 0.016095238 |
| Stanniocalcin-2 | STC2 | -3.388532911 | 0.331343873 | 0.011619048 |
| Nidogen-2 | NID2 | -3.385805198 | 0.323030675 | 0.012047619 |
| Lysosomal Pro-X carboxypeptidase | PRCP | -3.323272705 | 0.296399766 | 0.017333333 |
| Protocadherin alpha-C2 | PCDHAC2 | -3.286603315 | 0.317931513 | 0.015857143 |
| Tenascin-R | TNR | -3.257496561 | 0.320668592 | 0.012142857 |
| Cathepsin S | CTSS | -3.226269109 | 0.320972342 | 0.015666667 |
| Transgelin | TAGLN | -3.211675985 | 0.302170188 | 0.016904762 |
| Proteoglycan 4 | PRG4 | -3.181848935 | 0.289329648 | 0.019619048 |
| Transforming growth factor beta receptor type 3 | TGFBR3 | -3.140318053 | 0.289243658 | 0.019380952 |
| Carbonic anhydrase 4 | CA4 | -3.109602996 | 0.300735947 | 0.017 |
| Fibroblast growth factor receptor | FGFR2 | -3.054350649 | 0.286352759 | 0.01947619 |
| Integral membrane protein 2B | ITM2B | -3.042938096 | 0.293945375 | 0.017619048 |
| Endothelial protein C receptor | PROCR | -3.030934402 | 0.271515193 | 0.020428571 |
| C-C motif chemokine 14 | CCL14 | -3.027749402 | 0.29507715 | 0.017285714 |
| Dihydropteridine reductase | QDPR | -2.962807383 | 0.276973542 | 0.019904762 |
| Poliovirus receptor | PVR | -2.943588597 | 0.298442245 | 0.017142857 |
| Protein/nucleic acid deglycase DJ-1 | PARK7 | -2.913974217 | 0.259634845 | 0.020857143 |
| Complement component C8 gamma chain | C8G | -2.873356206 | 0.238384617 | 0.021761905 |
| Thrombospondin-2 | THBS2 | -2.867097923 | 0.274242039 | 0.020380952 |
| OX-2 membrane glycoprotein | CD200 | -2.845080512 | 0.185082266 | 0.029809524 |
| Multiple epidermal growth factor-like domains protein 8 | MEGF8 | -2.844053745 | 0.263737968 | 0.007619048 |
| Prosaposin receptor GPR37L1 | GPR37L1 | -2.801086221 | 0.152790267 | 0.029761905 |
| Immunoglobulin kappa variable 1-16 | IGKV1-16 | -2.744635514 | 0.166754983 | 0.030714286 |
| Eukaryotic translation initiation factor 2 subunit 3B | EIF2S3B | -2.654777391 | 0.122476715 | 0.033952381 |
| Cadherin EGF LAG seven-pass G-type receptor 2 | CELSR2 | -2.573299408 | 0.226133376 | 0.022285714 |
| Secretogranin-1 | CHGB | -2.552504744 | 0.16720634 | 0.021904762 |
| Epithelial discoidin domain-containing receptor 1 | DDR1 | -2.517567975 | 0.167282716 | 0.03052381 |
| Brain acid soluble protein 1 | BASP1 | -2.485378197 | 0.237088871 | 0.021809524 |
| Beta-galactoside alpha-2,6-sialyltransferase 2 | ST6GAL2 | -2.474132265 | 0.164098335 | 0.031142857 |
| Legumain | LGMN | -2.445262841 | 0.171111511 | 0.030190476 |
| Chitotriosidase-1 | CHIT1 | -2.444283554 | 0.167764212 | 0.03047619 |
| Cathepsin O | CTSO | -2.440262794 | 0.168166481 | 0.030428571 |
| Immunoglobulin heavy variable 1-2 | IGHV1-2 | -2.43029901 | 0.166559451 | 0.030761905 |
| Immunoglobulin heavy variable 3-13 | IGHV3-13 | -2.425952366 | 0.164246903 | 0.031095238 |
| Spectrin beta chain | SPTBN4 | -2.370889187 | 0.15760389 | 0.031761905 |
| Protein AHNAK2 | AHNAK2 | -2.35345711 | 0.158401641 | 0.031619048 |
| Protein NDRG2 | NDRG2 | -2.328153202 | 0.163578785 | 0.031238095 |
| Laminin subunit beta-2 | LAMB2 | -2.327467373 | 0.180199522 | 0.03 |
| Complement factor H-related protein 3 | CFHR3 | -2.325222628 | 0.15930271 | 0.03152381 |
| Histone H1.2 | H1-2 | -2.321843828 | 0.160586839 | 0.031428571 |
| Golgi apparatus protein 1, isoform CRA_c | GLG1 | -2.321016993 | 0.164673458 | 0.031 |
| Sortilin | SORT1 | -2.317759923 | 0.150170825 | 0.032142857 |
| Tissue alpha-L-fucosidase | FUCA1 | -2.317214761 | 0.110335499 | 0.034761905 |
| Immunoglobulin lambda variable 5-45 | IGLV5-45 | -2.279788085 | 0.158950014 | 0.031571429 |
| Ryanodine receptor 3 | RYR3 | -2.243037156 | 0.152863555 | 0.032047619 |
| Neurexin-3-beta | NRXN3 | -2.152274472 | 0.149621009 | 0.00352381 |
| Somatostatin | SST | -2.149101394 | 0.148217923 | 0.032380952 |
| Disintegrin and metalloproteinase domain-containing protein 11 | ADAM11 | -2.137351513 | 0.138136195 | 0.032904762 |
| Transmembrane protein 132C | TMEM132C | -2.079115186 | 0.091664585 | 0.03552381 |
| Protein FAM198B | GASK1B | -2.071126802 | 0.154662326 | 0.031952381 |
| Ectonucleotide pyrophosphatase/ phosphodiesterase family member 5 | ENPP5 | -2.066145556 | 0.135168902 | 0.033142857 |
| PITH domain-containing protein 1 | PITHD1 | -2.063803264 | 0.146547182 | 0.03247619 |
| Cadherin-10 | CDH10 | -2.054669789 | 0.148664552 | 0.032333333 |
| Stromal cell-derived factor 1 | CXCL12 | -2.029551097 | 0.145170627 | 0.032571429 |
| Nucleobindin-2 | NUCB2 | -2.021849837 | 0.141120811 | 0.032666667 |
| Receptor-type tyrosine-protein phosphatase kappa | PTPRK | -2.021718161 | 0.146872357 | 0.032428571 |
